# Interspecific variation in nectar and pollen reward among plant species commonly planted to support pollinators

**DOI:** 10.1093/aobpla/plag049

**Published:** 2026-10-12

**Authors:** Madeline Gregor, Wendy Lei, Adelaide Ergastolo, Sevan Suni

**Affiliations:** Department of Biology, The University of San Francisco, 2130 Fulton Street, San Francisco, CA 94117, United States; Department of Biology, The University of San Francisco, 2130 Fulton Street, San Francisco, CA 94117, United States; Department of Biology, The University of San Francisco, 2130 Fulton Street, San Francisco, CA 94117, United States; The Pollinator Partnership, 582 Market St Ste 1215, San Francisco, CA 94104, United States; Department of Biology, The University of San Francisco, 2130 Fulton Street, San Francisco, CA 94117, United States

**Keywords:** nectar, pollen, protein, pollinator, life history, perennial, flower, nutrition, *Scrophularia californica*, *Madia elegans*, *Epilobium canum*, common garden

## Abstract

Pollinators facilitate the reproduction of most wild plants. Declines in pollinator populations have led to interest in establishing floral enhancements to support them. However, knowledge about how food rewards vary across plant species planted to support pollinators remains scant. Using a greenhouse common garden, we quantified nectar volume, nectar sucrose, and pollen protein quantity for 11 species commonly planted to support pollinators in Mediterranean climates. We evaluated if variation was explained by life history and the potential for tradeoffs between nectar and pollen quality. There was high variation in nectar and moderate variation in pollen protein across species. The probability of producing nectar was higher for perennial plants. For flowers that contained nectar, there were no differences between annual and perennial plants in nectar traits. The species that produced the highest quality nectar (*Scrophularia californica*) produced pollen with the lowest protein amounts, and the species that produced the lowest quality nectar (*Madia elegans*) produced pollen with the highest protein amounts. There was no statistical association between pollen protein amounts and nectar traits across species. The nutrition pollinators obtain from foraging on most species studied here may come primarily in the form of carbohydrates or protein, but not both. However, one species (*Epilobium canum*) produced both high-quality nectar and pollen, so identifying others that produce similarly broad, high-quality rewards would be worthwhile. Our results also suggest that including perennial plants in pollinator gardens will facilitate pollinator acquisition of carbohydrates.

## Teaser

We quantified how food rewards to pollinators vary across plant species commonly planted to support pollinators in areas with Mediterranean climates. We found considerable variation among plant species in nectar and pollen quality, as well as indications that the probability of producing nectar is higher for perennial plants.

## Introduction

Pollinators facilitate the reproduction of 87% of all flowering plant species (Ollerton *et al.* 2011), and enhance seed set in at least 75% of crop varieties (Kearns *et al.* 1998). Declines in abundance have been documented for populations of many pollinator species (Potts *et al.* 2010), which have been linked to declines in populations of wild plants (Biesmeijer *et al.* 2006). Loss of pollinators also poses significant risk to economies worldwide, due to lower crop yields and increases in food prices (Murphy *et al.* 2022). Drivers of pollinator declines include pesticide use, changes in weather extremes, disease, poor nutrition, and habitat loss (Donkersley *et al.* 2014, Goulson *et al.* 2015, De Palma *et al.* 2016, Greenop *et al.* 2020). Habitat loss is of particular concern for pollinators because it can quickly eliminate nesting and food resources (Potts *et al.* 2010, Ollerton *et al.* 2014, Scheper *et al.* 2014, Baude *et al.* 2016) and also result in longer-term changes in genetic parameters that are important for population persistence (Schleimer and Frantz 2025). When habitat loss occurs due to urbanization, it is usually coupled with increases in the amount of impervious surface, invasive species, and heat island effects, all of which have additional negative consequences for biodiversity (Liang *et al.* 2023). Nevertheless, urban areas are increasingly being considered to be potentially important refuges for pollinators in human-dominated landscapes (Matteson and Langellotto 2010, Hall *et al.* 2017). Indeed, some studies have found that urban areas have higher pollinator species richness than nearby rural areas, with varying effects on abundance (Fortel *et al.* 2014, Baldock *et al.* 2015). Yet, other work found declines in bee diversity with increasing urbanization (Birdshire *et al.* 2020).

Efforts to reverse declines and stabilize pollinator populations often involve enhancing floral resources (Requier and Leonhardt 2020). Floral enhancements can include the establishment of hedgerows around agricultural fields (Morandin and Kremen 2013), or the planting of pollinator gardens in urban areas (Majewska and Altizer 2020). However, the effects of floral enhancements on pollinator abundance are mixed, with some studies finding positive associations between floral abundance and pollinator abundance or richness (Pardee and Philpott 2014, Quistberg *et al.* 2016, Bennett and Lovell 2019), and others finding no positive impacts (Glaum *et al.* 2017). It is possible that the contrasting results could be due to differences in the spatial scale or landscape types considered (Veddeler *et al.* 2006, Janvier *et al.* 2022), or the specific plants used in floral enhancements (Majewska and Altizer 2020).

Several considerations may increase the likelihood of success of resource augmentation efforts aimed to support pollinators. First, existing guides for pollinator garden establishment emphasize managing land in a way that facilitates pollinator reproduction, such as leaving bare ground for ground-nesting bees, planting caterpillar host plants to encourage egg-laying, or using boxes in which bees may nest (Black *et al.* 2016). Second, using plant mixes that result in phenological diversity in terms of flowering time may increase the likelihood that food resources are present to support species that are active at different times of the year (Tarrant *et al.* 2013). Third, larger gardens may lead to higher pollinator abundance and species diversity (Quistberg *et al.* 2016). Fourth, the extent to which plant mixtures contain native or non-native species can influence the ecological outcomes of pollinator gardens. Non-native plants are often planted in urban gardens due to their high availability and aesthetic appeal (Garbuzov and Ratnieks 2014). However, some horticulturally-derived plants that have been bred for aesthetic appeal have lost traits that allow them to support pollinators, such as high-quality nectar or reward accessibility (Comba 1999). In some cases, pollinators do not even visit non-native plants, possibly because they do not recognize them as potential food sources or cannot access the rewards (Corbet *et al.* 2001). Indeed, Fukase (2016) and Nabors *et al.* (2022) found increased pollinator activity for native relative to non-native plants. However, in contrast, Salisbury *et al.* (2015) found that pollinator gardens containing non-native plants supported more pollinators and Smith *et al.* (2006) found that the most important predictors of invertebrate species richness in urban gardens were plant structural heterogeneity and species richness rather than plant native status. Similarly, Garbuzov and Ratnieks (2014) found that while native plants attracted pollinators, many non-native plants were also attractive to pollinators. The extent to which native versus non-native plants support pollinators likely depends on whether they meet the nutritional requirements of different pollinator taxa (Jones and Rader 2022).

Plant species vary in the nutritional rewards provided to pollinators, such as the amount and sugar concentration of nectar produced (Fowler *et al.* 2016, Chalcoff *et al.* 2017, Liu *et al.* 2024), as well as the protein content of their pollen (Roulston *et al.* 2000, Roulston and Cane 2000, Vaudo *et al.* 2024a, Vaudo *et al.* 2024b). Resource partitioning among plant species is common in pollinator guilds (Jeavons *et al.* 2020, Ye *et al.* 2024). This may be due to pollinator-mediated selection that has driven the evolution of specialized floral forms that are accessible only to certain pollinators (Fenster *et al.* 2004). However, most plant species tend more towards generalization and are visited by several types of pollinators (Waser *et al.* 1996). For generalist plants, resource partitioning may be due to competition among pollinator species (Page and Williams 2023, Travis *et al.* 2025), variation among taxa in nutritional requirements (Jones and Rader 2022), or the parts of plants used as nutritional resources (Requier and Leonhardt 2020).

Most pollinators use nectar as a source of carbohydrates. The sources from which pollinators obtain amino acids and lipids vary, with bees typically obtaining the majority of their amino acids from pollen (Thakur and Nanda 2020), which is essential for the growth of their larvae (Herbert *et al.* 1977). Butterflies typically obtain amino acids from nectar (Erhardt and Rusterholz 1998, Mevi-Schütz and Erhardt 2005, Beck 2007), and hummingbirds often supplementing their diets with insect protein (Remsen 1986). Plants vary widely in the macronutrients provided to pollinators, which include carbohydrates in nectar (Liu *et al.* 2024) and lipids and protein in pollen (Vaudo *et al.* 2024b). Higher nutrient amounts in nectar and pollen have been linked to increased pollinator fitness (Roulston and Cane 2002, Cahenzli and Erhardt 2012, Di Pasquale *et al.* 2013), which may explain why flower visitors often show preferences for plants that have more concentrated nectar (Loo and Bitterman 1992, Roberts 1996, Cnaani *et al.* 2006, Fowler *et al.* 2016), or pollen with higher protein content (Vaudo *et al.* 2016). Therefore, understanding how the nutritional components of nectar and pollen vary among plant species is important to the success of habitat augmentation efforts that seek to support pollinator populations.

The overall aim of this study was to assess variation in reward to pollinators among plants commonly planted to support pollinators, and to evaluate drivers of such variation. Specifically, we quantified aspects of plant reward to pollinators for eleven plant species commonly planted to support pollinators in Mediterranean climate zones. We included both annual and perennial plants in our study, since life-history theory predicts that annual and perennial plant species may differ in the way they allocate energy to growth versus reproduction (Bazzaz *et al.* 1987). In particular, annual plants are expected to allocate more energy to reproduction than growth, which has been found empirically (Pitelka 1977, Primack 1979, Vico *et al.* 2016). However, work on whether life history mediates nutritional reward to pollinators is scant despite its potential importance to pollinator conservation (Russo *et al.* 2025). Therefore, our three aims were (i) to evaluate if nectar quality varies among species or with plant life history; (ii) to evaluate if pollen quality varies among species and or plant life history; and (iii) to investigate if there are associations between nectar quality and pollen quality across species.

## Materials and methods

### Species selection and greenhouse planting

We chose outcrossing plant species that span varying floral forms and colours and that have been documented to support pollinators in Mediterranean climates similar to where the current study took place in the San Francisco Bay Area of California (Dafni *et al.* 1988, Ogutcen *et al.* 2017, Lundin *et al.* 2019, Rollings and Goulson 2019, Nabors *et al.* 2022, Calflora.org). We worked with local horticulturalists to select appropriate species and obtained plants from Home Ground Habitats (Novato, CA), which is within the metropolitan area of San Francisco. Home Ground Habitats has the mission to promote habitat restoration and support pollinators through augmenting gardens with plants derived from wild-sourced seeds in Marin County, CA. We selected five annual and six perennial species that span various plant families, for which plants within species were the same age, and for which this was the first flowering event (Fig. 1). The annual plants were *Phacelia tanacetifolia* Benth. (‘Lacy phacelia’, Boraginaceae, native to the southwestern USA), *Gilia capitata* Sims (‘Globe gilia’, Polemoniaceae, native to the western USA), *Madia elegans* D. Don (‘Common madia’, Asteraceae, native to the western USA), *Clarkia rubicunda* Lindl. (‘Ruby chalice clarkia’, Onagraceae, endemic in California) and *Clarkia unguiculata* Lindl. (‘Elegant clarkia’, Onagraceae, endemic in California). The perennial species were *Epilobium canum* Greene (‘California fuchsia’, Onagraceae, native to the western USA), *Linaria purpurea* L. (‘Purple toadflax’, Plantaginaceae, native to Italy), *Calamintha nepeta* Willk. (‘Lesser calamint’, Lamiaceae, native to the Mediterranean region), *Salvia rosmarinus* Spenn. (‘Rosemary’, Lamiaceae, native to the Mediterranean region), *Scrophularia californica* Cham. & Schltdl. (‘California bee plant/figwort’, Scrophulariaceae, native to the western USA), and *Verbena lilacina* Greene (‘Cedros island verbena’, Verbenaceae, native to Baja California). We obtained a total of 102 individual plants (see Table 1 for sample sizes).

**Figure 1:**
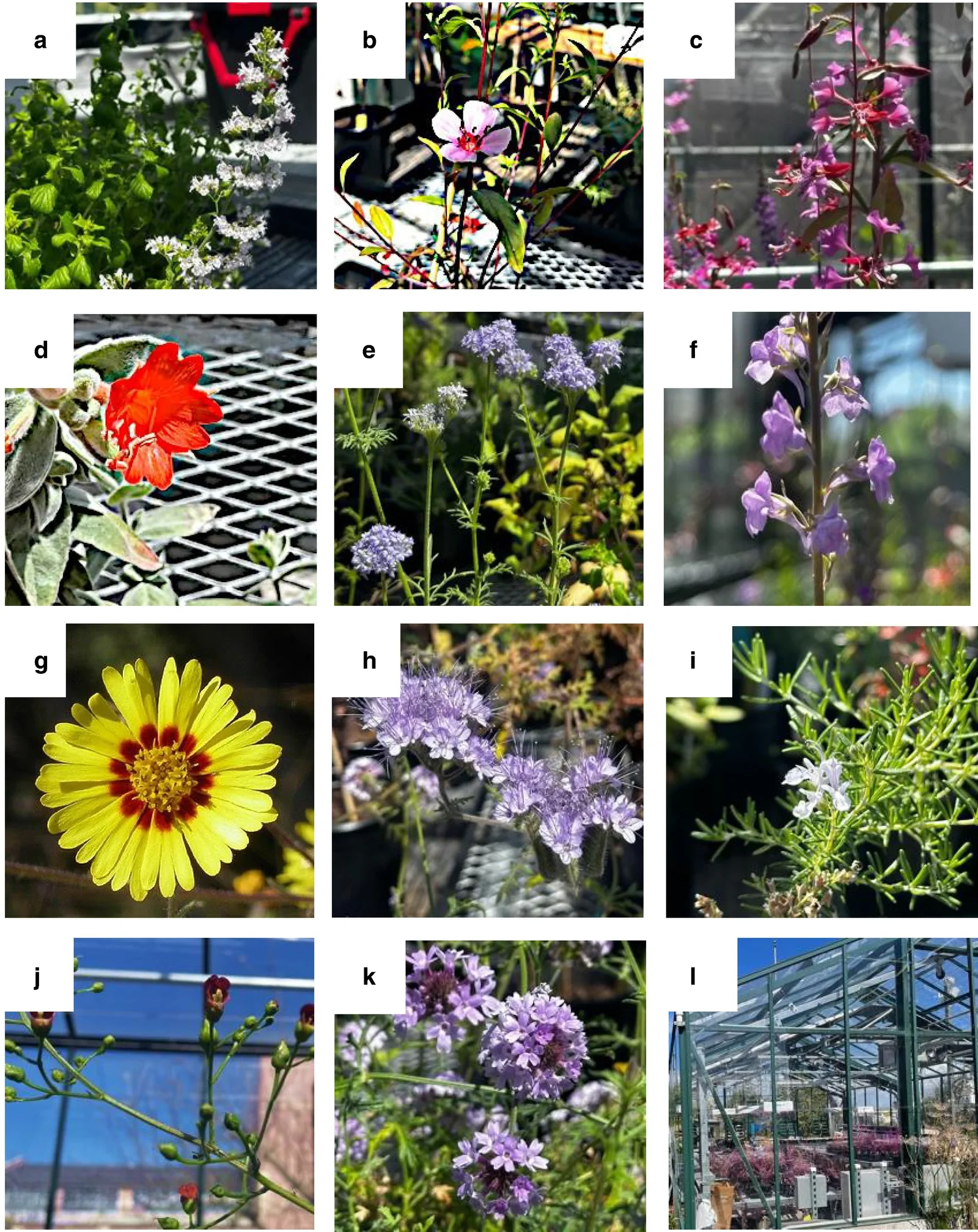
Plant species that were grown in the greenhouse common garden experiment. (a) *Calamintha nepeta* (Lamiaceae, Perennial); (b) *Clarkia rubicunda* (Onagraceae, Annual); (c) *Clarkia unguiculata* (Onagraceae, Annual); (d) *Epilobium canum* (Onagraceae, Perennial); (e): *Gilia capitata* (Polymoniaceae, Annual); (f) *Linaria purpurea* (Plantaginaceae, Perennial), (g) *Madia elegans* (Asteraceae, Annual, photo from https://en.wikipedia.org/wiki/Madia_elegans accessed on October 17, 2026); (h) *Phacelia tanacetifolia* (Boraginaceae, Annual); (i) *Salvia rosmarinus* (Lamiaceae, Perennial); (j) *Scrophularia californica* (Scrophulariaceae, Perennial); (k) *Verbena lilacina* (Verbenaceae, Perennial); (l) Greenhouse at the University of San Francisco, CA, USA.

**Table 1:** For each plant species used in the greenhouse common garden: the family, life history (life hist.), number of plant individuals included in experimental procedures (*N*), and mean nectar volumes, sucrose amounts, and pollen protein percentages with associated standard errors.

Plant species	Family	Life hist.	*N*	Nectar (μl)	Sucrose (mg)	Protein %
*Calamintha nepeta*	Lamiaceae	Perennial	8	0.23 ± 0.03	0.26 ± 0.03	---
*Clarkia rubicunda*	Onagraceae	Annual	4	---	---	8.91 ± 0.64
*Clarkia unguiculata*	Onagraceae	Annual	9	0.66 ± 0.10	0.69 ± 0.11	11.67 ± 0.54
*Epilobium canum*	Onagraceae	Perennial	5	3.33 ± 0.09	1.77 ± 0.27	13.0 ± 1.50
*Gilia capitata*	Polemoniaceae	Annual	11	0.07 ± 0.01	0.07 ± 0.01	---
*Linaria purpurea*	Plantaginaceae	Perennial	11 (3)	0.49 ± 0.06	0.56 ± 0.07	12.22 ± 1.56
*Madia elegans*	Asteraceae	Annual	13	0.03 ± 0.01	0.02 ± 0.00	12.96 ± 0.74
*Phacelia tanacetifolia*	Boraginaceae	Annual	12	0.20 ± 0.04	0.21 ± 0.04	---
*Salvia rosmarinus*	Lamiaceae	Perennial	8	0.43 ± 0.06	0.47 ± 0.06	---
*Scrophularia californica*	Scrophulariaceae	Perennial	10 (3)	3.25 ± 0.21	2.43 ± 0.16	6.23 ± 0.15
*Verbena lilacina*	Verbenaceae	Perennial	11	0.27 ± 0.04	0.17 ± 0.02	---

Ten species were used for assessment of nectar volume and six were used for assessment of protein. Sample sizes in parentheses reflect those used for pollen protein assessment if different from those used in nectar trait assessment. Nectar volume and sucrose amounts were measured for several flowers from each plant, and averages were generated by taking the mean across flowers within individuals and then taking the mean across individuals (see Supplementary Table S1). Pollen protein was assessed by pooling pollen from multiple flowers from the same individual and then assessing per cent protein in triplicate. Averages reflect means across replicates for each individual followed by taking the mean across individuals.

We obtained plants of all species except *E. canum* in 2024. *Epilobium canum* was not available in 2024 so we obtained it in 2025 (see below). After obtaining plants in 2024, we transferred individuals of each species into individual ½ gallon pots using soil provided by Home Ground Habitats, and placed plants in a greenhouse that was set to a 16-hour period of daylight, with a temperature range target of between 10 and 24°C, and a humidity target of 25%. We randomized the location of each plant with respect to its location in the greenhouse and watered all plants liberally every 3–4 days. We recorded the date on which each plant first flowered (see Supplementary Table S1). We quantified traits between the months of March and July in 2024. In 2025, we obtained *E. canum*, transferred individuals to ½ gallon pots using soil provided by Home Ground Habitats, and quantified traits between July and August (Supplementary Table S1).

### Nectar measurement

We sampled nectar from 5 to 11 individuals of ten species, which included multiple flowers per plant. Our final sample included a total of 545 flowers, which represented 2–6 flowers from each plant (mean flowers per plant = 5.42 ± 0.37; Table 1). We did not sample nectar from *C. rubicunda* because this species produced insufficient flowers for assessment of both nectar and pollen (see below). We took the following measures to facilitate consistency in measurements among plant individuals within each species. For *C. nepeta*, *E. canum*, *P. tanacetifolia*, and *R. officinalis*, we sampled flowers randomly from across the span of the plant, targeting flowers that were fully open and had not started to senesce. For *C. unguiculata*, *L. purpurea*, and *S. californica*, we sampled two flowers from the topmost, middle, and lower thirds of the plant, and we targeted flowers that were on different stems. For *G. capitata*, *M. elegans*, and *V. lilacina*, we targeted flowers on different umbels. We attempted to sample nectar from all individuals within a given species on the same days, and we repeated sampling of the same plants as more flowers opened (see Supplementary Table S1 for sampling details). We obtained nectar from plants between the hours of 10 a.m. and 5 p.m. and recorded the order that samples were taken on each day for each species.

Among the predominant sugars found in nectar (sucrose, glucose, and fructose), sucrose tends to dominate for most plant species in the New World (Baker and Baker 1983), those visited by insect pollinators (Dupont *et al.* 2004), and those visited by hummingbirds (Lotz and Schondube 2006). We therefore used sucrose percent and nectar volume as proxies for nectar quality, using previously described methods (Corbet *et al.* 2001, Suni *et al.* 2020, 2023). Briefly, we quantified nectar volume using 5 μl microcapillary tubes (Drummond Scientific Company, Broomall, PA) and digital callipers. We quantified the percent sucrose using a handheld refractometer (Bellingham & Stanley, Ltd., Lawrenceville, GA). The refractometer measures % Brix, which is the grams of total dissolved solid material in nectar per 100 g of total solution (Corbet 2003). We then converted the % Brix to milligrams of sucrose present in the nectar (Bolten *et al.* 1979).

### Pollen protein assessment

We used the percent protein in pollen as a proxy for pollen quality, as in Vaudo *et al.* (2024a, 2024b). We did this for five of the species for which nectar quality was assessed (*C. unguiculata*, *E. canum*, *L. purpurea*, *M. elegans*, and *S. californica*), as well as for one additional species, *C. rubicunda* (Table 1). We chose these species because they produced amounts of pollen sufficient for the assessment of protein amounts based on the Bradford Assay Protocol (Bradford 1976, Vaudo *et al.* 2024a, Ergastolo 2025). To implement the Bradford Assay, we used a Pierce Bradford Plus Protein Assay Kit with G-250 dye in a colorimetric reagent for the detection and quantitation of total protein (ThermoFisher Scientific, Waltham, MA). The Bradford protein assay is used to measure the concentration of total protein in a sample and is robust to different amounts of starting material as long as samples are sufficiently diluted so that absorbance values fall on the linear part of the curve relating absorbance to concentration (Zor and Selinger 1996).

To measure the percent protein in pollen, we removed anthers from several flowers per plant using sterilized tweezers and stored them at −20 °C prior to analysis. The number of flowers from which we removed anthers varied among species and with anther size, such that we used 2–5 flowers for *C. unguiculata and C. rubicunda*, 3 flowers for *E. canum*, 8–15 flowers for *L. purpurea* and *S. californica*, and 20–40 flowers of *M. elegans*. For each plant individual, we pooled samples from all sampled flowers and then dried them in separate uncapped 1.5 ml centrifuge tubes in an Isotemp oven (Fisher Scientific) at 40°C for 72 hours. After drying, we vortexed the anthers to dislodge the pollen, transferred the pollen to clean 1.5 ml centrifuge tubes, and weighed the pollen. Then, we added 100 µl NaOH to the tubes containing the pollen, vortexed for 30 seconds, centrifuged, and pulsed on the vortex before allowing the pollen to sit at room temperature for 30 minutes. Meanwhile, we removed the Bradford reagent from the refrigerator (4°C) and allowed it to reach room temperature for 30 minutes. After 30 minutes, pollen solutions with concentrations above 4 mg/ml were further diluted to under that threshold before proceeding to the next step to ensure that absorbance values would fall on the linear part of the absorbance–concentration curve. We then added 392 µl Bradford reagent to a clean 1.5 ml centrifuge tube. We vortexed the diluted pollen solution and pipetted up and down and added 8 µl of the pollen solution to the tube containing the Bradford reagent. We vortexed and centrifuged this mixture and then pipetted three 100 µl aliquots of the mixture into a 96-well Black Flat Bottom Microplate (Corning, Corning, NY) so that we could run samples in triplicate. We popped any bubbles using a sterilized needle before allowing the plate to sit at room temperature for 10 minutes. We determined the absorbance of each well at 595 nm using a Tecan plate reader. We then determined the protein concentrations of each sample using a standard curve generated Albumin Standards provided in the Pierce Bradford Plus Protein Assay Kit.

### Statistical modelling

#### General approach

We ran generalized linear mixed models (GLMMs) implemented in R (R Core Team 2024), using the glmmTMB package (Brooks *et al.* 2017). We examined residuals to determine model fit using the package DHARMa (Hartig 2024). We evaluated the significance of fixed factors using likelihood ratio tests on nested models with and without the factor of interest implemented using the anova command. In the results section, we report chi-square statistics and degrees of freedom from likelihood ratio tests. We provide model coefficients (Est = estimate; CI = 95% confidence interval) generated using the package sjPlot (Lüdecke *et al*. 2021), because sjPlot provides back-transformed coefficients on the response scale and is therefore useful applying models with non-Gaussian error structures. We also used sjPlot to generate marginal and conditional *R*^2^ values, which are useful for evaluating the amount of the amount of variance explained by fixed effects alone (marginal) and the amount of variance explained by both fixed and random effects (conditional). We also report the ranges of trait values along with raw means and standard errors. We used Claude (Anthropic 2026) to provide general guidance on appropriate error structures for GLMMs given specific data types and to assist with interpretation of zero-inflated model output (see below).

### Aim 1: assess if nectar quality varies among species or with plant life history

To determine if nectar volume and sucrose amount vary among plant species, we used a dataset that contained nectar volume and sucrose amounts for multiple flowers from each of 98 plant individuals, across ten species and two life-history categories. We ran separate GLMMs with nectar volume or sucrose amount as the dependent variable using a Tweedie distribution and log link function, and with flower position and plant individual as random effects. To determine if nectar volume or sucrose amount vary with life history (annual or perennial), we ran separate GLMMs with zero-inflated (ZI) Gamma error distributions and log link functions, and with flower position and plant individual as random effects. In the results section we report model coefficients pertaining to both the ZI and count portions of the model, which reflect the probability of producing nectar (ZI portion; same for both nectar volume and sucrose because plants that produced no nectar also produced no nectar sucrose), or the volume of nectar or amount of sucrose for flowers that did produce sucrose (count portions).

### Aim 2: assess if pollen protein content varies among species or with plant life history

To determine if pollen protein content varies among species or with life history, we used a dataset that contained 111 observations comprised of three replicates of per cent protein for 37 plant individuals, across six plant species and two life-history categories. We ran separate GLMMs with Gaussian error distributions and log link functions. We included plant species or life history as the independent variable, and plant individual as a random effect.

### Aim 3: evaluate associations between nectar quality and pollen quality, and determine if they vary with plant life history

We examined if there is a relationship between the nectar and pollen quality using a dataset that was made from the datasets used to evaluate Aims 1 & 2. This dataset contained average nectar volume, average nectar sucrose, and average per cent protein for each plant individual for which both nectar and protein amounts were quantified (33 plant individuals representing five species and two life-history categories). We ran GLMMs with zero-inflated (ZI) Gamma error distributions and log link functions with nectar volume or sucrose amount as the dependent variable. We included percent protein, plant life history, and their interaction as independent variables, and plant species as a random effect. We also used this same model structure to evaluate if nectar volume or sucrose amount varied with life history when controlling for the effect of protein percentages.

## Results

Nectar volume ranged from 0 to 4.50 µl within flowers across plant individuals (Table 1). There was substantial variation among species in nectar volume (Tables 1 and 2; Fig. 2a). The mean nectar volume for annual plants was 0.24 ± 0.14 µl and for perennial plants it was 1.33 ± 0.14 µl. Annual plants had a lower probability of producing nectar (Odds ratio reflecting the probability of not producing nectar for perennial relative to annual = 0.028; Table 2). Among plants that did produce nectar, there was no difference between annual and perennial plants in the amount produced (Table 2). Nectar sucrose ranged from 0 to 3.08 mg across plant individuals (Table 1). Plant species varied substantially in their nectar sucrose (Tables 1 and 2; Fig. 2b). The mean nectar sucrose for annual plants was 0.25 ± 0.15, and the mean for perennial plants was 0.94 ± 0.38. Among flowers that did produce sucrose, there was no difference between annual and perennial plants (Table 2).

**Figure 2:**
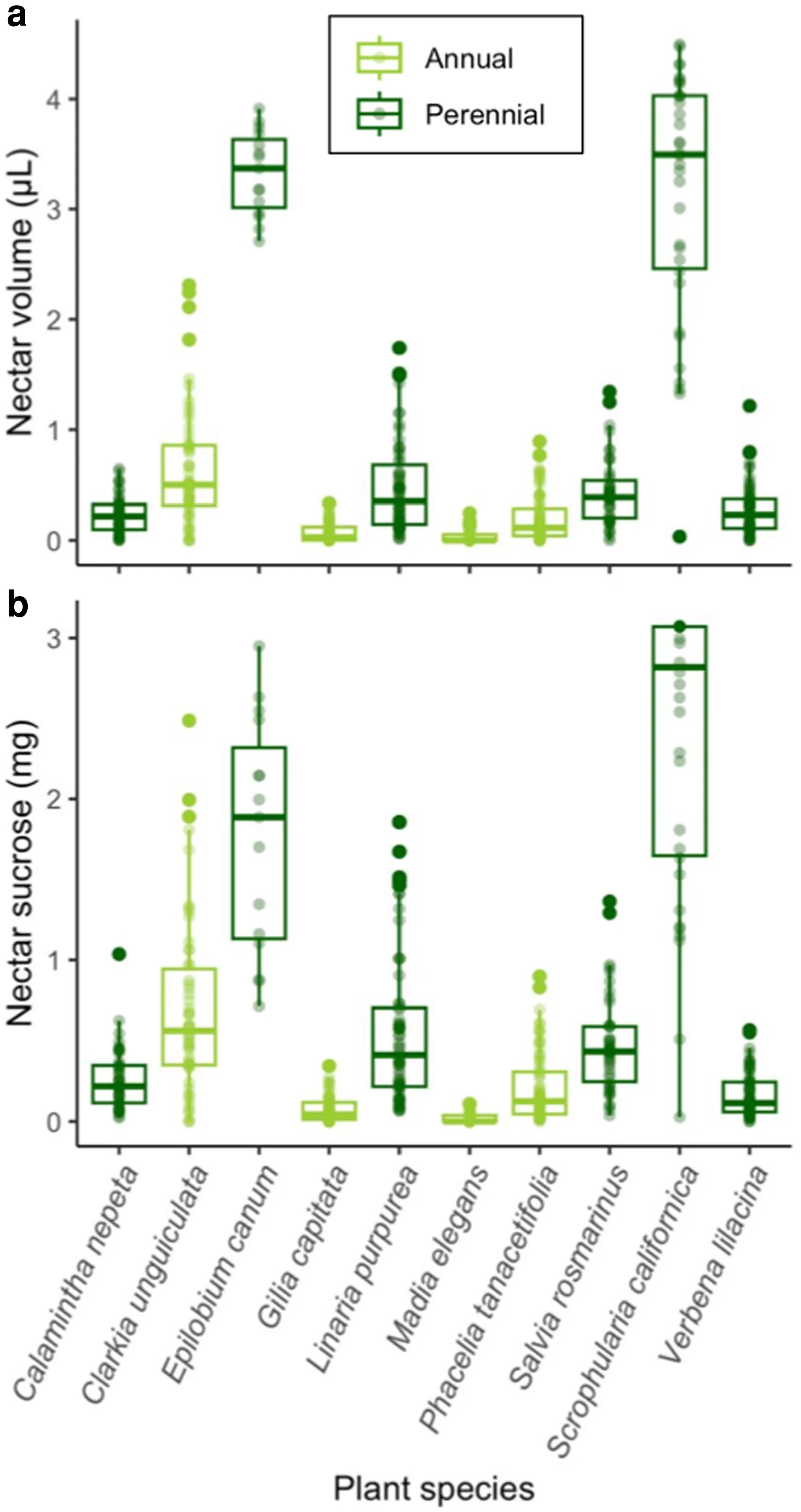
The volume of nectar (panel a) and amount of sucrose (panel b) found in flowers of plant species commonly planted to support pollinators and grown in a greenhouse common garden. Plots are boxplots and points represent nectar volumes of individual flowers. Colours reflect plant life history, with light green representing annual plant species and dark green representing perennial plant species.

**Table 2:** Results from generalized linear mixed models (GLMMs) assessing drivers of variation in nectar and protein quality across plant species commonly planted to support pollinators.

Trait	Family, link	Random	R^2^	Predictors	Effect size	CI	Interp.	χ^2^	df	*P*
Volume (µl)	Tweedie Log	Flower Ind.	M: 0.805 C: 0.863	Species	---	---	Ratio	120.960	6	<.001
Sucrose (mg)	Tweedie Log	Flower Ind.	M: 0.845 C: 0.912	Species	---	---	Ratio	110.280	6	<.001
Volume (µl)	ZI Gamma Log	Flower Ind. Species	M: 0.194 C: 0.705	ZI: Life hist. C: Life hist.	0.028 3.542	0.007–0.116 0.801–15.672	Odds ratio Ratio	68.597 2.31	1 1	<.001 .128
Sucrose (mg)	ZI Gamma Log	Flower Ind. Species	M: 0.155 C: 0.814	ZI: Life hist. C: Life hist.	0.028 3.117	0.007–0.116 0.594–16.345	Odds ratio Ratio	68.597 1.594	1 1	<.001 0.207
Protein (%)	Gaussian Log	Flower Ind.	M: 0.050 C: 0.081	Species	---	---	Ratio	32.691	5	<.001
Protein (%)	Gaussian Log	Flower Ind. Species	M: 0.003 C: 0.076	Life hist.	−0.115	−0.298–0.068	Ratio	1.496	1	.221
Volume (µl)	ZI Gamma Log	Species	M: 0.462 C: 0.878	ZI: Life hist. C: Protein*Life hist. C: Protein C: Life hist.	0.000 0.926 1.043 30.16	0.000—∞ 0.748–1.145 0.903–1.203 1.240–733.249	Odds ratio Ratio	0.827 0.502 0.022 3.755	1 1 1 1	.363 .479 .882 .053
Sucrose (mg)	ZI Gamma Log	Species	M: 0.452 C: 0.901	ZI: Life hist. C: Protein*Life hist. C: Protein C: Life hist.	0.000 1.002 1.015 11.829	0.000—∞ 0.834–1.204 0.902–1.141 0.597–234.237	Odds ratio Ratio	1.685 0.001 0.111 3.540	1 1 1 1	.194 .982 .739 .060

For each dependent variable (Trait), the model family with error structure and link function specified (Family, Link), random effects included (Random; Flower = the position of the flower on the plant; Ind. = plant individual; Species = plant species), conditional (C) and marginal (M) R^2^ values, fixed effects included (Predictors; Species = plant species, Life hist = life history; ZI or C = zero-inflated or count portion of zero-inflated GLMM), back-transformed model coefficients (Effect size); 95% confidence intervals (CI), an interpretation of the effect size (Interp.), and results from likelihood ratio tests on nested models used to assess significance of fixed factors and their interactions (χ^2^ values, degrees of freedom (df), and *P*-values (*P*)). Note: post-hoc pairwise comparisons among plant species were not performed, and effect size for models assessing effects of life history on traits reflect values for perennials relative to annuals.

The percent protein in pollen ranged from 6.01 to 18.03 across plant individuals (Table 1) and varied across species (Tables 1 and 2; Fig. 3). The mean percent protein for annual plants was 11.18 ± 1.2 and the mean for perennial plants was 10.49 ± 2.15, and there was no statistical difference between annual and perennial plants in percent protein in pollen (Table 2). There was no difference between annual and perennial plants in the relationship between the amount of protein in pollen and nectar volume (Table 2; Fig. 4a) or nectar sucrose (Table 2; Fig. 4b). There was no relationship between the amount of protein in pollen and the probability of nectar production (Table 2), nectar volume (Table 2; Fig. 4a), or nectar sucrose amount (Fig. 4b). When controlling for the effect of protein, there was a non-significant trend towards higher protein amounts being associated with higher nectar volumes (Table 2) and higher sucrose amounts (Table 2).

**Figure 3:**
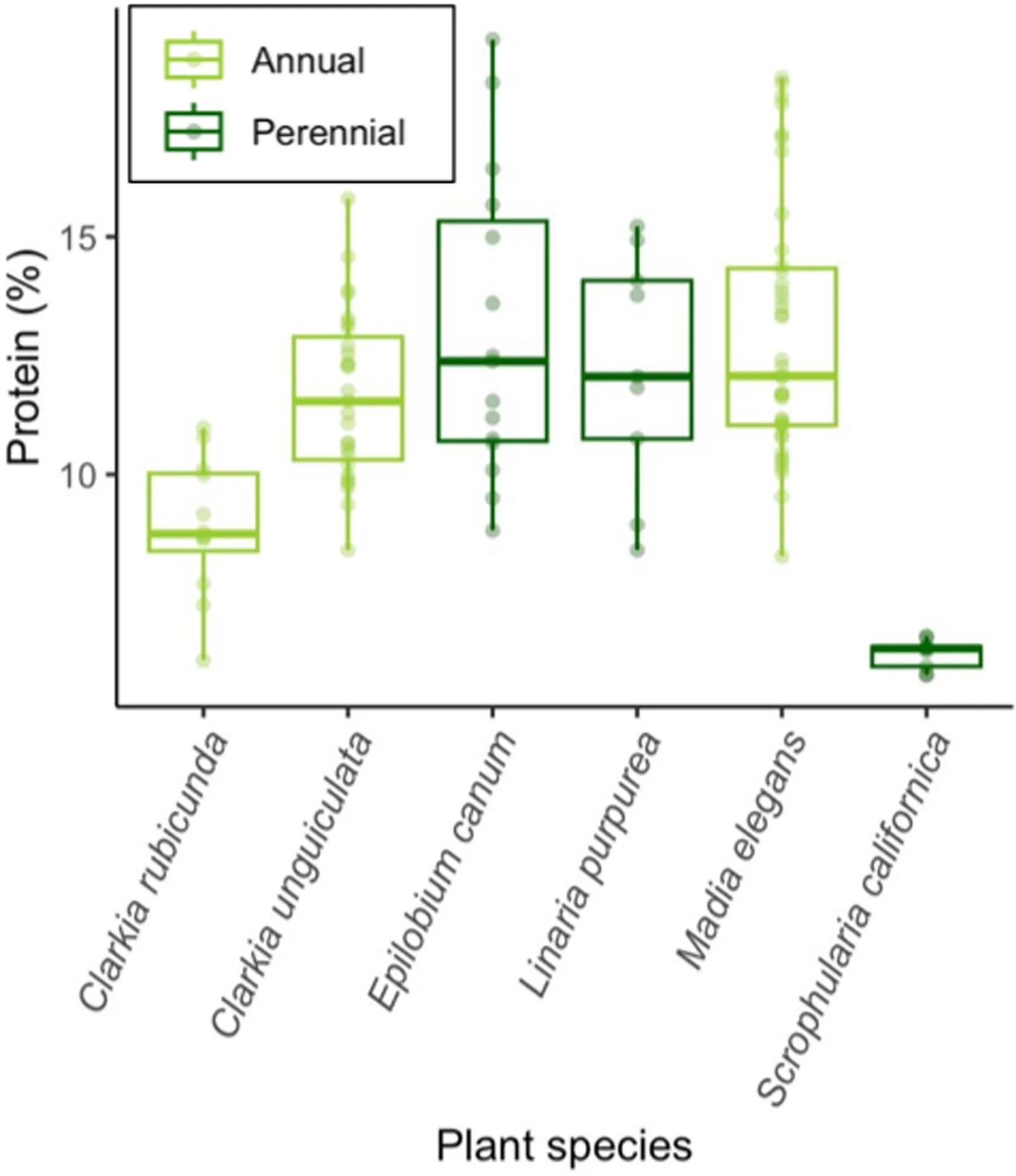
The percent protein found in pollen of flowers of plant species commonly planted to support pollinators and grown in a greenhouse common garden. Plots are boxplots and points represent nectar volumes of individual flowers. Colours reflect plant life history, with light green representing annual plant species and dark green representing perennial plant species.

**Figure 4:**
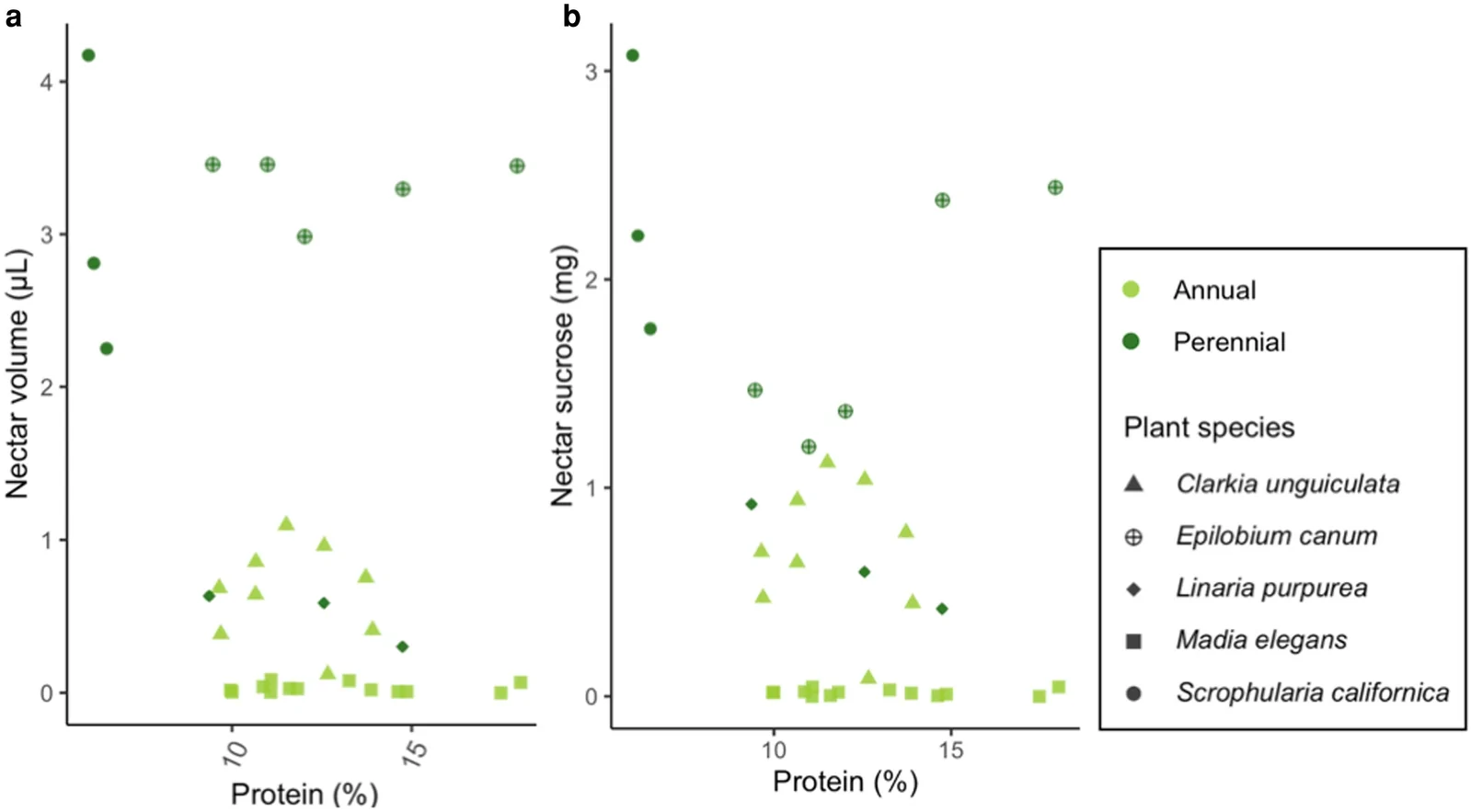
The relationship between the percent protein in pollen and the nectar volume (panel a) and amount of sucrose present in nectar (panel b) for plant species commonly planted to support pollinators and grown in a greenhouse common garden. Points represent individual plants, colours represent plant life history, with light green representing annual plant species and dark green representing perennial plant species, and shapes represent the individual plant species.

## Discussion

Nectar and pollen nutritional resources are critical for pollinators, yet most studies have examined them separately (Parachnowitsch *et al.* 2019). Furthermore, studies that have investigated nectar and pollen quality for the same plant species have tended to lack a statistical examination of how the traits may vary jointly (Dmitruk *et al.* 2024, Sulborska-Różycka et al. 2025). Our integrated analysis of nectar and pollen quality for plants commonly planted to support pollinators reveals substantial differences among species, as well as variation with life history in the probability of nectar production. There were no apparent tradeoffs between nectar and pollen quality across species, nor differences in the relationship between nectar and pollen quality for plants with different life histories. Rather, species tended to occupy distinct parts of the nectar and protein quality landscape with some producing large amounts of high-quality nectar but low protein quality (*S. californica*), others producing both high quality nectar and high protein amounts (*E. canum*), and the majority producing relatively low-quality nectar with varying pollen protein amounts.

Nectar volumes and sucrose amounts varied over two orders of magnitude among the plant species examined, suggesting that some may better provision pollinators with water and carbohydrates. In particular, *S. californica* produced the most nectar as well as nectar with the highest sucrose content. The high-nectar quality of *S. californica* may help explain the results of previous work showing that in coastal California, bumble bee density was positively associated with floral abundance of *S. californica* (Thomson 2016). *Scrophularia californica* is capable of dynamic nectar secretion, such that removal of nectar stimulates additional nectar production (Morris 2007). Given that plant species vary in whether they exhibit responsive nectar secretion (Galetto and Bernardello 1993, Castellanos *et al.* 2002, Luo *et al.* 2014), the high-quality nectar produced by *S. californica*, as well as the ability to replenish nectar once visited make this species a logical choice to include in pollinator gardens.

In contrast to *S. californica*, *M. elegans* produced the lowest volume of nectar, as well as nectar with the lowest sucrose content. The low carbohydrate rewards produced by *M. elegans* are consistent with past work that found only moderate visitation to *M. elegans* relative to other species used by home gardeners to support pollinators (Anderson *et al.* 2022). *Madia elegans* has glandular trichomes that produce a sticky exudate that entrap insects (Pearse *et al.* 2024). It is possible that tradeoffs between allocation of sugars to defence versus pollinator reward could explain the low investment of *M. elegans* into nectar quality (Jacobsen and Raguso 2018). It is also worth noting that *M. elegans* may produce carbohydrate rewards to pollinators that were not examined in the current study. Some species in Asteraceae have low sucrose to hexose ratios in their nectar, such that the hexose sugars glucose and fructose are the main carbohydrates present (Torres and Galetto 2002, Liu *et al.* 2024). Despite the low nectar quality of *M. elegans*, its pollen contained protein amounts that were moderate to high among the species studied here. In addition, pollen from some plants in Asteraceae have been found to contain relatively high amounts of lipids (Vaudo *et al.* 2024a), which are also important nutritional resources that pollen-foraging insects get from pollen (Furse *et al.* 2023). It is therefore likely that pollinators can obtain useful nutritional resources from *M. elegans* in the form of protein or lipids, but the low nectar volumes found in this study suggest other plants would better provision them with carbohydrates.

Relative to variation in nectar volume, variation in pollen protein percentages among species was lower and varied approximately two-fold across species. The percentages we document here are within the range reported in past work, which have been shown to vary more than three-fold across species and be phylogenetically conserved to some extent (Buchmann 1986, Roulston and Cane 2000, Ruedenauer *et al.* 2019, Vaudo *et al.* 2020, Vaudo *et al.* 2024a). Our results support the notion that pollen protein percentages are phylogenetically conserved, as the percentages we document are largely consistent with relative differences found among families in other work. Among the families that were examined both in this study and in that of Vaudo *et al.* (2020), species from Scrophulariaceae produced pollen with the lowest protein percentages, and species in Plantaginaceae produced pollen with high protein amounts relative to other taxa. It is also worth noting that although plant species differed significantly in pollen protein percentages, we also found substantial variation within species. Other work that explored intraspecific variation in pollen protein quantity found more than two-fold variation within a single species (Dmitruk *et al.* 2024), as well as variation among sites (Vaudo *et al.* 2024b). Those studies were conducted using field-collected pollen, so the variation was likely driven at least in part by environmental effects. Indeed, Vaudo *et al.* (2024) showed that higher climatic water deficits (CWD) were associated with higher pollen protein concentrations. However, there was high variability in the relationship between CWD and pollen protein concentration, suggesting additional mechanisms drove protein concentrations. Given that the plants for which we measured pollen quality were grown in a climate-controlled greenhouse, our findings suggest the potential for substantial intraspecific genetic or epigenetic variation in alleles that contribute to pollen protein quantity. The high variation suggests there is potential for habitat managers or horticulturalists to select plants that produce high protein quantities for use in floral enhancements.

The variation we found in plant provisioning of nectar rewards to pollinators contrasts somewhat with life-history theory, which addresses how limited resources are allocated to growth versus reproduction. Traditional assumptions of life-history theory include that annual plants should allocate proportionally more fixed carbon to reproduction than perennials, all else being equal (Bazzaz *et al.* 1987). Consistent with theory, annual plants have been found to allocate more energy to seed production than perennial plants (Pitelka 1977, Primack 1979, Vico *et al.* 2016). It might also be expected that annuals would invest more energy in nectar production to encourage visitation, pollen transfer by pollinators, and subsequent seed production. However, the probability of nectar production was far lower for the annual plants examined in this study. Furthermore, of the 10 species for which nectar volume and sucrose amounts were examined, the top seven included only one annual species, with the other three annuals falling to the bottom in terms of nectar quality. Similarly, other work using experimental meadows found that those that contained perennial plant mixes produced 20 times as much nectar as those with annual plant mixes (Hicks *et al.* 2016).

It is possible that the high provisioning of nectar by some perennials and none of the annuals in our study could be due to costs of nectar production. There is evidence that the production of nectar by plants is costly (Pyke and Ren 2023), and that allocation of energy to nectar production can reduce growth and the ability to produce seeds (Pyke 1991). Since annual plants must complete their lifecycles quickly, they tend to have higher relative growth rates than perennial plants (Lundgren and Des Marais 2020), which may constrain the amount of energy they can allocate to nectar relative to perennial plants. However, there is little experimental evidence that tradeoffs exist between nectar quality and traits that reflect plant size, such as above-ground biomass, root biomass, or number of flowers (Pyke and Ren 2023). Furthermore, since greater pollen protein amounts are associated with higher seed production (Schweizer and Ries 1969), a negative relationship between nectar and pollen quality may be expected in annual plants. However, we found no evidence of tradeoffs between nectar and pollen quality, although we note that for perennials *S. californica* and *L. purpurea*, individuals that produced the most nectar also had the lowest pollen protein amounts and vice versa (but this observation was based on only three individual plants per species). It is also worth noting that our pollen analysis included only six species, so the investigation of whether there is a relationship between nectar and pollen quality was also based on that restricted subset. Therefore, more work that includes additional species as well as larger sample sizes within species will help clarify if tradeoffs exist in how energy is allocated to nectar versus other aspects of plant growth and development.

Another reason that perennial plants had a higher probability of producing nectar could be due to differences in the strength of pollinator-mediated selection across life-history categories. Nectar volume and sucrose content are heritable traits that can respond to selection by pollinators (Parachnowitsch *et al.* 2019). Perennial plants experience selection by pollinators over several years, while annual plants experience selection by pollinators only during a single growing season. Abiotic conditions can exert selection on floral traits that oppose pollinator-mediated selection (Sletvold 2019). It is possible that other selective forces acting on nectar traits, such as environmental fluctuations across years, dampen responses to selection by pollinators on nectar traits for annual plants. In contrast, perennial plants experience selection by pollinators over a longer time-period, so yearly environmental fluctuations may have less of an effect on responses to selection for perennial plants. We therefore speculate that the longer time periods over which perennial plants experience selection by pollinators may result in stronger pollinator-mediated selection on nectar traits for perennial plants. Whether or not this could be responsible for the high nectar amounts and high sugar content seen in some perennial plants would be a worthwhile future research direction.

While data on reward quality for most species in our dataset are scant, we can draw comparisons with past studies on *P. tanacetifolia* and *S. rosmarinus*. *Phacelia tanacetifolia* has long been considered to be an important source of nutrition from both nectar and pollen for bees (reviewed in Żarczyński *et al.* 2025). Past measurements of nectar sucrose ranged from 1.0 to 4.5 mg per flower (Cîrlig 2021), which are lower than the amounts we found in the current study. The nectar volumes we found for *S. rosmarinus* were also on the lower end of the range documented in past work (Jaworski *et al.* 2022). We speculate that these discrepancies could be due to differences in soil nutrients, since the amount of soil nitrogen can affect nectar production (David *et al.* 2019), or they could be due to variation in reward quality among plants from different geographic areas (Lanza *et al.* 1995, Farkas *et al.* 2012, Suni *et al.* 2023). It would also be worthwhile for future studies to include measurements of morphological and growth data in future analyses, since nectar production has been shown to vary with other plant traits such as the number of open flowers (Biernaskie and Cartar 2004), and energetic investment in pollen has been shown to vary with plant size (Delesalle and Mazer 2009).

## Conclusions

To our knowledge, this is the first study that has examined associations between nectar and pollen quality and that has contextualized measurements in the context of plant life history for multiple annual and perennial plants simultaneously. Our results suggest that for most plant species in this study, the nutritional benefits that pollinators might obtain from foraging on them may come primarily in the form of carbohydrates or protein, but not both. However, some plant species clearly produce both high-quality nectar and pollen (e.g. *E. canum* in this study), so identifying these species and including them in floral enhancements would likely benefit pollinators. In addition, this research demonstrated high intraspecific variation in reward to pollinators for some species. For perennial plants *S. californica* and *L. purpurea*, average nectar volume and sucrose amounts decreased as average pollen protein amounts increased. The small sample sizes for these species prevented any within-species statistical analyses, so it would be interesting to characterize the relationship between nectar and pollen quality for these species using larger sample sizes and investigate possible drivers if relationships were found, including costs of producing nectar, energetic tradeoffs, or pollinator-mediated selection. Finally, the variation we found across plant species also provides insight into which plants may best support pollinators that forage primarily for nectar or pollen. It would be worthwhile to investigate if pollinators that visit *M. elegans* forage primarily for pollen and not nectar, given the low-quality nectar and higher-quality pollen produced by this plant species.

## Supplementary Material

plag049_Supplementary_Data

## Data Availability

The data and code used in analyses are available online: [dataset] Data and code for ‘Nectar and pollen reward to pollinators vary interspecifically and with life history for plant species commonly planted to support pollinators’, Zenodo online data repository, DOI:10.5281/zenodo.20837630

## AI assistance

Claude (Anthropic 2026) was used to provide general guidance on appropriate error structures for GLMMs given specific data types and to assist with interpreting model output, including zero-inflation and count components of zero-inflated models, and back-transformation of coefficients. All statistical models, analyses, and interpretations were performed independently by the authors, without the use of AI.
