# Supplementary material for: Interspecific variation in nectar and pollen reward among plant species commonly planted to support pollinators
Source: AoB Plants. 2026 Oct 12;18(5):plag049. doi: 10.1093/aobpla/plag049 (PMC13700956; doi:10.1093/aobpla/plag049)
Supplement: Supplementary file 1 [file plag049_supplementary_data.pdf]

Supplementary information for: Interspecific variation in nectar and pollen reward among plant species commonly planted to support pollinators

Table S1. For each individual flower for which nectar volume and sucrose were measured, the order in which it was measured, the specimen number of the individual plant (Spec.), the they tray it was in in the greenhouse, the species, the life history (Life hist.), the date it first flowered (Flowering date), the date that flowers were sampled for nectar assessment (Trait date), the position of the flower that was measured (Position), the number of the flower that was measured (Flower), the nectar volume of that flower (Vol.) in microliters, and the amount of sucrose in that flower (Sucrose) in milligrams.

Order	Spec.	Tray	Species	Life hist.	Flowering date	Trait date	Position	Flower	Vol.	Sucrose
247	2	1	*Clarkia unguiculata*	Annual	04.07.2024	4/14/24	Bottom	1	0.38	0.37
248	2	1	*Clarkia unguiculata*	Annual	04.07.2024	4/17/24	Bottom	2	0.38	0.34
250	2	1	*Clarkia unguiculata*	Annual	04.07.2024	4/24/24	Middle	4	1.17	1.11
249	2	1	*Clarkia unguiculata*	Annual	04.07.2024	4/24/24	Middle	3	0.23	0.24
252	2	1	*Clarkia unguiculata*	Annual	04.07.2024	4/27/24	Top	6	1.11	1.30
251	2	1	*Clarkia unguiculata*	Annual	04.07.2024	4/27/24	Top	5	0.84	0.79
253	18	6	*Clarkia unguiculata*	Annual	04.11.2024	4/14/24	Bottom	1	0.50	0.59
254	18	6	*Clarkia unguiculata*	Annual	04.11.2024	4/17/24	Bottom	2	0.50	0.59
255	18	6	*Clarkia unguiculata*	Annual	04.11.2024	4/24/24	Middle	3	0.52	0.49
256	18	6	*Clarkia unguiculata*	Annual	04.11.2024	4/24/24	Middle	4	0.86	0.85
257	18	6	*Clarkia unguiculata*	Annual	04.11.2024	4/27/24	Top	5	0.68	0.59
258	18	6	*Clarkia unguiculata*	Annual	04.11.2024	4/27/24	Top	6	0.79	0.75
259	20	7	*Clarkia unguiculata*	Annual	04.14.2024	4/14/24	Bottom	1	0.52	0.46
260	20	7	*Clarkia unguiculata*	Annual	04.14.2024	4/24/24	Bottom	2	0.49	0.46
262	20	7	*Clarkia unguiculata*	Annual	04.14.2024	4/24/24	Middle	4	1.21	1.33
261	20	7	*Clarkia unguiculata*	Annual	04.14.2024	4/24/24	Middle	3	1.82	1.89
264	20	7	*Clarkia unguiculata*	Annual	04.14.2024	4/27/24	Top	6	0.68	0.83
263	20	7	*Clarkia unguiculata*	Annual	04.14.2024	4/27/24	Top	5	0.43	0.67
277	31	11	*Clarkia unguiculata*	Annual	04.27.2024	4/30/24	Bottom	1	0.00	0.00
278	31	11	*Clarkia unguiculata*	Annual	04.27.2024	4/30/24	Bottom	2	0.00	0.00
279	31	11	*Clarkia unguiculata*	Annual	04.27.2024	4/30/24	Middle	3	0.23	0.17
280	31	11	*Clarkia unguiculata*	Annual	04.27.2024	4/30/24	Middle	4	0.00	0.01
281	31	11	*Clarkia unguiculata*	Annual	04.27.2024	5/5/24	Top	5	0.29	0.13
282	31	11	*Clarkia unguiculata*	Annual	04.27.2024	5/5/24	Top	6	0.20	0.20
289	40	14	*Clarkia unguiculata*	Annual	04.19.2024	4/24/24	Bottom	1	0.66	0.52
290	40	14	*Clarkia unguiculata*	Annual	04.19.2024	4/24/24	Bottom	2	0.61	0.60
291	40	14	*Clarkia unguiculata*	Annual	04.19.2024	4/27/24	Middle	3	0.83	0.87
292	40	14	*Clarkia unguiculata*	Annual	04.19.2024	4/27/24	Middle	4	1.46	1.68
293	40	14	*Clarkia unguiculata*	Annual	04.19.2024	4/27/24	Top	5	0.50	0.70
294	40	14	*Clarkia unguiculata*	Annual	04.19.2024	4/30/24	Top	6	0.45	0.33
301	45	15	*Clarkia unguiculata*	Annual	04.09.2024	4/14/24	Bottom	1	0.41	0.48
302	45	15	*Clarkia unguiculata*	Annual	04.09.2024	4/17/24	Bottom	2	0.37	0.35
303	45	15	*Clarkia unguiculata*	Annual	04.09.2024	4/24/24	Middle	3	2.24	1.99

304	45	15	*Clarkia unguiculata*	Annual	04.09.2024	4/24/24	Middle	4	2.31	2.49
305	45	15	*Clarkia unguiculata*	Annual	04.09.2024	4/27/24	Top	5	0.87	0.97
306	45	15	*Clarkia unguiculata*	Annual	04.09.2024	4/27/24	Top	6	0.37	0.44
313	55	19	*Clarkia unguiculata*	Annual	04.04.2024	4/14/24	Bottom	1	0.11	0.07
314	55	19	*Clarkia unguiculata*	Annual	04.04.2024	4/17/24	Bottom	2	0.09	0.16
315	55	19	*Clarkia unguiculata*	Annual	04.04.2024	4/24/24	Middle	3	1.02	1.06
316	55	19	*Clarkia unguiculata*	Annual	04.04.2024	4/24/24	Middle	4	0.45	0.67
317	55	19	*Clarkia unguiculata*	Annual	04.04.2024	4/27/24	Top	5	0.54	0.53
318	55	19	*Clarkia unguiculata*	Annual	04.04.2024	4/27/24	Top	6	0.10	0.35
319	79	27	*Clarkia unguiculata*	Annual	04.17.2024	4/17/24	Bottom	1	0.04	0.09
320	79	27	*Clarkia unguiculata*	Annual	04.17.2024	4/24/24	Bottom	2	2.11	1.81
322	79	27	*Clarkia unguiculata*	Annual	04.17.2024	4/24/24	Middle	4	1.26	1.06
321	79	27	*Clarkia unguiculata*	Annual	04.17.2024	4/24/24	Middle	3	1.40	1.27
324	79	27	*Clarkia unguiculata*	Annual	04.17.2024	4/27/24	Top	6	0.15	0.66
323	79	27	*Clarkia unguiculata*	Annual	04.17.2024	4/27/24	Top	5	0.81	1.33
325	93	31	*Clarkia unguiculata*	Annual	04.25.2024	4/30/24	Bottom	1	0.31	0.36
326	93	31	*Clarkia unguiculata*	Annual	04.25.2024	4/30/24	Bottom	2	0.94	0.97
327	93	31	*Clarkia unguiculata*	Annual	04.25.2024	4/30/24	Middle	3	0.51	0.54
328	93	31	*Clarkia unguiculata*	Annual	04.25.2024	4/30/24	Middle	4	0.34	0.39
329	93	31	*Clarkia unguiculata*	Annual	04.25.2024	4/30/24	Top	5	0.36	0.36
330	93	31	*Clarkia unguiculata*	Annual	04.25.2024	4/30/24	Top	6	0.01	0.06
485	1	1	*Gilia capitata*	Annual	04.11.2024	5/5/24	Umbel_1	1	0.20	0.27
486	1	1	*Gilia capitata*	Annual	04.11.2024	5/5/24	Umbel_2	2	0.20	0.13
487	1	1	*Gilia capitata*	Annual	04.11.2024	5/5/24	Umbel_3	3	0.00	0.06
488	1	1	*Gilia capitata*	Annual	04.11.2024	5/5/24	Umbel_4	4	0.12	0.06
489	1	1	*Gilia capitata*	Annual	04.11.2024	5/5/24	Umbel_5	5	0.09	0.08
490	1	1	*Gilia capitata*	Annual	04.11.2024	5/5/24	Umbel_6	6	0.11	0.09
491	6	2	*Gilia capitata*	Annual	04.17.2024	5/5/24	Umbel_1	1	0.04	0.04
492	6	2	*Gilia capitata*	Annual	04.17.2024	5/5/24	Umbel_2	2	0.00	0.00
493	6	2	*Gilia capitata*	Annual	04.17.2024	5/5/24	Umbel_3	3	0.00	0.00
494	6	2	*Gilia capitata*	Annual	04.17.2024	5/5/24	Umbel_4	4	0.00	0.00
495	6	2	*Gilia capitata*	Annual	04.17.2024	5/5/24	Umbel_5	5	0.00	0.00
496	6	2	*Gilia capitata*	Annual	04.17.2024	5/5/24	Umbel_6	6	0.00	0.00
497	53	18	*Gilia capitata*	Annual	04.14.2024	5/5/24	Umbel_1	1	0.00	0.00
498	53	18	*Gilia capitata*	Annual	04.14.2024	5/5/24	Umbel_2	2	0.00	0.00
499	53	18	*Gilia capitata*	Annual	04.14.2024	5/5/24	Umbel_3	3	0.10	0.03
500	53	18	*Gilia capitata*	Annual	04.14.2024	5/5/24	Umbel_4	4	0.05	0.03
501	53	18	*Gilia capitata*	Annual	04.14.2024	5/5/24	Umbel_5	5	0.00	0.03
502	53	18	*Gilia capitata*	Annual	04.14.2024	5/5/24	Umbel_6	6	0.16	0.12
503	76	26	*Gilia capitata*	Annual	04.17.2024	5/5/24	Umbel_1	1	0.15	0.15

504	76	26	*Gilia capitata*	Annual	04.17.2024	5/5/24	Umbel_2	2	0.00	0.00
505	76	26	*Gilia capitata*	Annual	04.17.2024	5/5/24	Umbel_3	3	0.26	0.24
506	76	26	*Gilia capitata*	Annual	04.17.2024	5/5/24	Umbel_4	4	0.19	0.24
507	76	26	*Gilia capitata*	Annual	04.17.2024	5/5/24	Umbel_5	5	0.00	0.00
508	76	26	*Gilia capitata*	Annual	04.17.2024	5/5/24	Umbel_6	6	0.00	0.00
509	77	26	*Gilia capitata*	Annual	04.19.2024	5/5/24	Umbel_1	1	0.04	0.03
510	77	26	*Gilia capitata*	Annual	04.19.2024	5/5/24	Umbel_2	2	0.00	0.00
511	77	26	*Gilia capitata*	Annual	04.19.2024	5/5/24	Umbel_3	3	0.22	0.16
512	77	26	*Gilia capitata*	Annual	04.19.2024	5/5/24	Umbel_4	4	0.02	0.04
513	77	26	*Gilia capitata*	Annual	04.19.2024	5/5/24	Umbel_5	5	0.20	0.10
514	77	26	*Gilia capitata*	Annual	04.19.2024	5/5/24	Umbel_6	6	0.03	0.01
515	78	26	*Gilia capitata*	Annual	04.17.2024	5/5/24	Umbel_1	1	0.12	0.15
516	78	26	*Gilia capitata*	Annual	04.17.2024	5/5/24	Umbel_2	2	0.08	0.12
517	78	26	*Gilia capitata*	Annual	04.17.2024	5/5/24	Umbel_3	3	0.07	0.08
518	78	26	*Gilia capitata*	Annual	04.17.2024	5/5/24	Umbel_4	4	0.01	0.04
519	78	26	*Gilia capitata*	Annual	04.17.2024	5/5/24	Umbel_5	5	0.06	0.09
520	78	26	*Gilia capitata*	Annual	04.17.2024	5/5/24	Umbel_6	6	0.03	0.17
521	89	30	*Gilia capitata*	Annual	04.17.2024	5/5/24	Umbel_1	1	0.06	0.03
522	89	30	*Gilia capitata*	Annual	04.17.2024	5/5/24	Umbel_2	2	0.00	0.03
523	89	30	*Gilia capitata*	Annual	04.17.2024	5/5/24	Umbel_3	3	0.12	0.12
524	89	30	*Gilia capitata*	Annual	04.17.2024	5/5/24	Umbel_4	4	0.06	0.03
525	89	30	*Gilia capitata*	Annual	04.17.2024	5/5/24	Umbel_5	5	0.00	0.05
526	89	30	*Gilia capitata*	Annual	04.17.2024	5/5/24	Umbel_6	6	0.09	0.07
533	97	33	*Gilia capitata*	Annual	04.21.2024	5/5/24	Umbel_1	1	0.01	0.03
534	97	33	*Gilia capitata*	Annual	04.21.2024	5/5/24	Umbel_2	2	0.13	0.10
535	97	33	*Gilia capitata*	Annual	04.21.2024	5/5/24	Umbel_3	3	0.00	0.01
536	97	33	*Gilia capitata*	Annual	04.21.2024	5/5/24	Umbel_4	4	0.07	0.04
537	97	33	*Gilia capitata*	Annual	04.21.2024	5/5/24	Umbel_5	5	0.00	0.02
538	97	33	*Gilia capitata*	Annual	04.21.2024	5/5/24	Umbel_6	6	0.04	0.04
539	101	34	*Gilia capitata*	Annual	04.17.2024	5/5/24	Umbel_1	1	0.00	0.02
540	101	34	*Gilia capitata*	Annual	04.17.2024	5/5/24	Umbel_2	2	0.27	0.25
541	101	34	*Gilia capitata*	Annual	04.17.2024	5/5/24	Umbel_3	3	0.33	0.34
542	101	34	*Gilia capitata*	Annual	04.17.2024	5/5/24	Umbel_4	4	0.00	0.01
543	101	34	*Gilia capitata*	Annual	04.17.2024	5/5/24	Umbel_5	5	0.00	0.00
544	101	34	*Gilia capitata*	Annual	04.17.2024	5/5/24	Umbel_6	6	0.02	0.09
545	103	35	*Gilia capitata*	Annual	04.17.2024	5/5/24	Umbel_1	1	0.00	0.00
546	103	35	*Gilia capitata*	Annual	04.17.2024	5/5/24	Umbel_2	2	0.14	0.14
547	103	35	*Gilia capitata*	Annual	04.17.2024	5/5/24	Umbel_3	3	0.00	0.10
548	103	35	*Gilia capitata*	Annual	04.17.2024	5/5/24	Umbel_4	4	0.00	0.00
549	103	35	*Gilia capitata*	Annual	04.17.2024	5/5/24	Umbel_5	5	0.00	0.04

550	103	35	*Gilia capitata*	Annual	04.17.2024	5/5/24	Umbel_6	6	0.00	0.01
551	107	36	*Gilia capitata*	Annual	04.14.2024	5/5/24	Umbel_1	1	0.00	0.00
552	107	36	*Gilia capitata*	Annual	04.14.2024	5/5/24	Umbel_2	2	0.00	0.04
553	107	36	*Gilia capitata*	Annual	04.14.2024	5/5/24	Umbel_3	3	0.25	0.20
554	107	36	*Gilia capitata*	Annual	04.14.2024	5/5/24	Umbel_4	4	0.01	0.13
555	107	36	*Gilia capitata*	Annual	04.14.2024	5/5/24	Umbel_5	5	0.19	0.21
556	107	36	*Gilia capitata*	Annual	04.14.2024	5/5/24	Umbel_6	6	0.19	0.15
557	3	1	*Madia elegans*	Annual	07.14.2024	7/17/24	Umbel_1	1	0.09	0.07
558	3	1	*Madia elegans*	Annual	07.14.2024	7/17/24	Umbel_1	2	0.00	0.00
559	3	1	*Madia elegans*	Annual	07.14.2024	7/17/24	Umbel_2	3	0.01	0.01
560	3	1	*Madia elegans*	Annual	07.14.2024	7/17/24	Umbel_3	4	0.00	0.00
561	3	1	*Madia elegans*	Annual	07.14.2024	7/17/24	Umbel_4	5	0.00	0.03
562	3	1	*Madia elegans*	Annual	07.14.2024	7/17/24	Umbel_5	6	0.00	0.00
563	4	2	*Madia elegans*	Annual	07.14.2024	7/1/24	Umbel_1	1	0.00	0.00
564	4	2	*Madia elegans*	Annual	07.14.2024	7/1/24	Umbel_1	2	0.00	0.00
565	4	2	*Madia elegans*	Annual	07.14.2024	7/1/24	Umbel_1	3	0.00	0.00
566	4	2	*Madia elegans*	Annual	07.14.2024	7/1/24	Umbel_2	4	0.00	0.00
567	4	2	*Madia elegans*	Annual	07.14.2024	7/1/24	Umbel_2	5	0.00	0.00
568	4	2	*Madia elegans*	Annual	07.14.2024	7/1/24	Umbel_2	6	0.00	0.00
569	11	4	*Madia elegans*	Annual	06.30.2024	6/30/24	Umbel_1	1	0.15	0.11
573	11	4	*Madia elegans*	Annual	06.30.2024	6/30/24	Umbel_1	5	0.00	0.00
570	11	4	*Madia elegans*	Annual	06.30.2024	6/30/24	Umbel_2	2	0.11	0.07
574	11	4	*Madia elegans*	Annual	06.30.2024	6/30/24	Umbel_2	6	0.08	0.04
571	11	4	*Madia elegans*	Annual	06.30.2024	6/30/24	Umbel_3	3	0.06	0.02
572	11	4	*Madia elegans*	Annual	06.30.2024	6/30/24	Umbel_4	4	0.12	0.04
575	12	4	*Madia elegans*	Annual	06.30.2024	6/30/24	Umbel_1	1	0.00	0.00
576	12	4	*Madia elegans*	Annual	06.30.2024	6/30/24	Umbel_1	2	0.00	0.00
577	12	4	*Madia elegans*	Annual	06.30.2024	6/30/24	Umbel_2	3	0.02	0.06
578	12	4	*Madia elegans*	Annual	06.30.2024	6/30/24	Umbel_2	4	0.13	0.06
579	12	4	*Madia elegans*	Annual	06.30.2024	6/30/24	Umbel_3	5	0.09	0.02
580	12	4	*Madia elegans*	Annual	06.30.2024	6/30/24	Umbel_3	6	0.00	0.00
581	33	11	*Madia elegans*	Annual	07.11.2024	7/11/24	Umbel_1	1	0.09	0.06
582	33	11	*Madia elegans*	Annual	07.11.2024	7/11/24	Umbel_1	2	0.08	0.05
583	33	11	*Madia elegans*	Annual	07.11.2024	7/11/24	Umbel_2	3	0.07	0.05
584	33	11	*Madia elegans*	Annual	07.11.2024	7/11/24	Umbel_2	4	0.00	0.00
585	33	11	*Madia elegans*	Annual	07.11.2024	7/11/24	Umbel_3	5	0.11	0.06
586	33	11	*Madia elegans*	Annual	07.11.2024	7/11/24	Umbel_3	6	0.06	0.05
587	41	14	*Madia elegans*	Annual	07.11.2024	7/11/24	Umbel_1	1	0.00	0.00
588	41	14	*Madia elegans*	Annual	07.11.2024	7/11/24	Umbel_1	2	0.00	0.00
589	41	14	*Madia elegans*	Annual	07.11.2024	7/11/24	Umbel_2	3	0.00	0.00

590	41	14	*Madia elegans*	Annual	07.11.2024	7/11/24	Umbel_2	4	0.17	0.02
591	41	14	*Madia elegans*	Annual	07.11.2024	7/11/24	Umbel_3	5	0.00	0.00
592	41	14	*Madia elegans*	Annual	07.11.2024	7/11/24	Umbel_3	6	0.00	0.00
593	42	14	*Madia elegans*	Annual	07.14.2024	7/28/24	Umbel_1	1	0.00	0.00
594	42	14	*Madia elegans*	Annual	07.14.2024	7/28/24	Umbel_1	2	0.00	0.00
595	42	14	*Madia elegans*	Annual	07.14.2024	7/28/24	Umbel_2	3	0.00	0.05
596	42	14	*Madia elegans*	Annual	07.14.2024	7/28/24	Umbel_2	4	0.00	0.00
597	42	14	*Madia elegans*	Annual	07.14.2024	7/28/24	Umbel_3	5	0.00	0.00
598	42	14	*Madia elegans*	Annual	07.14.2024	7/28/24	Umbel_4	6	0.04	0.02
599	52	18	*Madia elegans*	Annual	07.14.2024	7/28/24	Umbel_1	1	0.00	0.00
600	52	18	*Madia elegans*	Annual	07.14.2024	7/28/24	Umbel_1	2	0.00	0.00
601	52	18	*Madia elegans*	Annual	07.14.2024	7/28/24	Umbel_1	3	0.00	0.00
602	52	18	*Madia elegans*	Annual	07.14.2024	7/28/24	Umbel_2	4	0.03	0.00
603	52	18	*Madia elegans*	Annual	07.14.2024	7/28/24	Umbel_2	5	0.00	0.00
604	52	18	*Madia elegans*	Annual	07.14.2024	7/28/24	Umbel_2	6	0.00	0.00
605	54	18	*Madia elegans*	Annual	07.14.2024	7/28/24	Umbel_1	1	0.03	0.01
606	54	18	*Madia elegans*	Annual	07.14.2024	7/28/24	Umbel_1	2	0.00	0.05
607	54	18	*Madia elegans*	Annual	07.14.2024	7/28/24	Umbel_1	3	0.00	0.00
608	54	18	*Madia elegans*	Annual	07.14.2024	7/28/24	Umbel_1	4	0.00	0.00
609	54	18	*Madia elegans*	Annual	07.14.2024	7/28/24	Umbel_1	5	0.00	0.00
610	54	18	*Madia elegans*	Annual	07.14.2024	7/28/24	Umbel_1	6	0.00	0.05
611	61	21	*Madia elegans*	Annual	07.01.2024	7/17/24	Umbel_1	1	0.05	0.04
612	61	21	*Madia elegans*	Annual	07.01.2024	7/17/24	Umbel_2	2	0.00	0.00
613	61	21	*Madia elegans*	Annual	07.01.2024	7/17/24	Umbel_3	3	0.00	0.00
614	61	21	*Madia elegans*	Annual	07.01.2024	7/17/24	Umbel_4	4	0.00	0.00
615	61	21	*Madia elegans*	Annual	07.01.2024	7/17/24	Umbel_5	5	0.06	0.05
616	61	21	*Madia elegans*	Annual	07.01.2024	7/17/24	Umbel_6	6	0.05	0.03
617	64	22	*Madia elegans*	Annual	06.26.2024	7/2/24	Umbel_1	1	0.00	0.00
618	64	22	*Madia elegans*	Annual	06.26.2024	7/2/24	Umbel_1	2	0.01	0.04
619	64	22	*Madia elegans*	Annual	06.26.2024	7/2/24	Umbel_2	3	0.00	0.00
620	64	22	*Madia elegans*	Annual	06.26.2024	7/2/24	Umbel_2	4	0.00	0.00
621	64	22	*Madia elegans*	Annual	06.26.2024	7/2/24	Umbel_3	5	0.00	0.02
622	64	22	*Madia elegans*	Annual	06.26.2024	7/2/24	Umbel_3	6	0.10	0.03
623	83	28	*Madia elegans*	Annual	07.08.2024	7/17/24	Umbel_1	1	0.00	0.00
624	83	28	*Madia elegans*	Annual	07.08.2024	7/17/24	Umbel_1	2	0.25	0.08
625	83	28	*Madia elegans*	Annual	07.08.2024	7/17/24	Umbel_1	3	0.12	0.05
626	83	28	*Madia elegans*	Annual	07.08.2024	7/17/24	Umbel_1	4	0.06	0.04
627	83	28	*Madia elegans*	Annual	07.08.2024	7/17/24	Umbel_1	5	0.05	0.02
628	83	28	*Madia elegans*	Annual	07.08.2024	7/17/24	Umbel_1	6	0.00	0.00
629	85	29	*Madia elegans*	Annual	06.30.2024	7/2/24	Umbel_1	1	0.00	0.00

630	85	29	*Madia elegans*	Annual	06.30.2024	7/2/24	Umbel_1	2	0.00	0.00
631	85	29	*Madia elegans*	Annual	06.30.2024	7/2/24	Umbel_2	3	0.00	0.00
632	85	29	*Madia elegans*	Annual	06.30.2024	7/2/24	Umbel_2	4	0.05	0.02
633	85	29	*Madia elegans*	Annual	06.30.2024	7/2/24	Umbel_3	5	0.00	0.00
634	85	29	*Madia elegans*	Annual	06.30.2024	7/2/24	Umbel_4	6	0.00	0.00
127	7	3	*Phacelia tanacetifolia*	Annual	04.04.2024	4/5/24	NA	1	0.16	0.15
128	7	3	*Phacelia tanacetifolia*	Annual	04.04.2024	4/5/24	NA	2	0.02	0.05
129	7	3	*Phacelia tanacetifolia*	Annual	04.04.2024	4/7/24	NA	3	0.07	0.11
130	7	3	*Phacelia tanacetifolia*	Annual	04.04.2024	4/7/24	NA	4	0.15	0.12
131	7	3	*Phacelia tanacetifolia*	Annual	04.04.2024	4/7/24	NA	5	0.05	0.03
132	7	3	*Phacelia tanacetifolia*	Annual	04.04.2024	4/7/24	NA	6	0.02	0.04
133	19	7	*Phacelia tanacetifolia*	Annual	04.04.2024	4/5/24	NA	1	0.07	0.11
134	19	7	*Phacelia tanacetifolia*	Annual	04.04.2024	4/5/24	NA	2	0.11	0.15
135	19	7	*Phacelia tanacetifolia*	Annual	04.04.2024	4/7/24	NA	3	0.06	0.10
136	19	7	*Phacelia tanacetifolia*	Annual	04.04.2024	4/7/24	NA	4	0.22	0.21
137	19	7	*Phacelia tanacetifolia*	Annual	04.04.2024	4/7/24	NA	5	0.12	0.12
138	19	7	*Phacelia tanacetifolia*	Annual	04.04.2024	4/7/24	NA	6	0.77	0.83
139	23	8	*Phacelia tanacetifolia*	Annual	04.09.2024	4/5/24	NA	1	0.07	0.02
140	23	8	*Phacelia tanacetifolia*	Annual	04.09.2024	4/5/24	NA	2	0.03	0.03
141	23	8	*Phacelia tanacetifolia*	Annual	04.09.2024	4/7/24	NA	3	0.13	0.14
142	23	8	*Phacelia tanacetifolia*	Annual	04.09.2024	4/7/24	NA	4	0.11	0.02
143	23	8	*Phacelia tanacetifolia*	Annual	04.09.2024	4/7/24	NA	5	0.65	0.56
144	23	8	*Phacelia tanacetifolia*	Annual	04.09.2024	4/7/24	NA	6	0.03	0.08
145	27	9	*Phacelia tanacetifolia*	Annual	04.04.2024	4/5/24	NA	1	0.01	0.01
146	27	9	*Phacelia tanacetifolia*	Annual	04.04.2024	4/5/24	NA	2	0.04	0.02
147	27	9	*Phacelia tanacetifolia*	Annual	04.04.2024	4/7/24	NA	3	0.14	0.12
148	27	9	*Phacelia tanacetifolia*	Annual	04.04.2024	4/7/24	NA	4	0.08	0.12
149	27	9	*Phacelia tanacetifolia*	Annual	04.04.2024	4/7/24	NA	5	0.00	0.05
150	27	9	*Phacelia tanacetifolia*	Annual	04.04.2024	4/7/24	NA	6	0.07	0.03
151	34	12	*Phacelia tanacetifolia*	Annual	04.04.2024	4/5/24	NA	1	0.09	0.07
152	34	12	*Phacelia tanacetifolia*	Annual	04.04.2024	4/5/24	NA	2	0.41	0.42
153	34	12	*Phacelia tanacetifolia*	Annual	04.04.2024	4/7/24	NA	3	0.41	0.35
154	34	12	*Phacelia tanacetifolia*	Annual	04.04.2024	4/7/24	NA	4	0.29	0.22
155	34	12	*Phacelia tanacetifolia*	Annual	04.04.2024	4/7/24	NA	5	0.25	0.27
156	34	12	*Phacelia tanacetifolia*	Annual	04.04.2024	4/7/24	NA	6	0.35	0.32
157	37	13	*Phacelia tanacetifolia*	Annual	When purchased	4/5/24	NA	1	0.02	0.05
158	37	13	*Phacelia tanacetifolia*	Annual	When purchased	4/5/24	NA	2	0.16	0.17
159	37	13	*Phacelia tanacetifolia*	Annual	When purchased	4/7/24	NA	3	0.09	0.09
160	37	13	*Phacelia tanacetifolia*	Annual	When purchased	4/7/24	NA	4	0.56	0.51
161	37	13	*Phacelia tanacetifolia*	Annual	When purchased	4/7/24	NA	5	0.24	0.29

Supplementary information for: Interspecific variation in nectar and pollen reward among plant species commonly planted to support pollinators

162	37	13	*Phacelia tanacetifolia*	Annual	When purchased	4/7/24	NA	6	0.60	0.61
163	38	13	*Phacelia tanacetifolia*	Annual	04.07.2024	4/7/24	NA	1	0.02	0.02
164	38	13	*Phacelia tanacetifolia*	Annual	04.07.2024	4/7/24	NA	2	0.01	0.02
165	38	13	*Phacelia tanacetifolia*	Annual	04.07.2024	4/7/24	NA	3	0.04	0.06
166	38	13	*Phacelia tanacetifolia*	Annual	04.07.2024	4/9/24	NA	4	0.20	0.15
167	38	13	*Phacelia tanacetifolia*	Annual	04.07.2024	4/9/24	NA	5	0.04	0.04
168	38	13	*Phacelia tanacetifolia*	Annual	04.07.2024	4/17/24	NA	6	0.41	0.38
169	70	24	*Phacelia tanacetifolia*	Annual	04.04.2024	4/5/24	NA	1	0.05	0.03
170	70	24	*Phacelia tanacetifolia*	Annual	04.04.2024	4/5/24	NA	2	0.04	0.08
171	70	24	*Phacelia tanacetifolia*	Annual	04.04.2024	4/7/24	NA	3	0.16	0.17
172	70	24	*Phacelia tanacetifolia*	Annual	04.04.2024	4/7/24	NA	4	0.33	0.17
173	70	24	*Phacelia tanacetifolia*	Annual	04.04.2024	4/7/24	NA	5	0.28	0.38
174	70	24	*Phacelia tanacetifolia*	Annual	04.04.2024	4/7/24	NA	6	0.20	0.49
175	72	24	*Phacelia tanacetifolia*	Annual	04.04.2024	4/5/24	NA	1	0.02	0.02
176	72	24	*Phacelia tanacetifolia*	Annual	04.04.2024	4/5/24	NA	2	0.23	0.30
177	72	24	*Phacelia tanacetifolia*	Annual	04.04.2024	4/7/24	NA	3	0.08	0.13
178	72	24	*Phacelia tanacetifolia*	Annual	04.04.2024	4/7/24	NA	4	0.00	0.08
179	72	24	*Phacelia tanacetifolia*	Annual	04.04.2024	4/7/24	NA	5	0.07	0.09
180	72	24	*Phacelia tanacetifolia*	Annual	04.04.2024	4/7/24	NA	6	0.61	0.69
181	84	28	*Phacelia tanacetifolia*	Annual	04.04.2024	4/5/24	NA	1	0.08	0.10
182	84	28	*Phacelia tanacetifolia*	Annual	04.04.2024	4/5/24	NA	2	0.16	0.14
183	84	28	*Phacelia tanacetifolia*	Annual	04.04.2024	4/7/24	NA	3	0.16	0.15
184	84	28	*Phacelia tanacetifolia*	Annual	04.04.2024	4/7/24	NA	4	0.58	0.57
185	84	28	*Phacelia tanacetifolia*	Annual	04.04.2024	4/7/24	NA	5	0.40	0.46
186	84	28	*Phacelia tanacetifolia*	Annual	04.04.2024	4/7/24	NA	6	0.40	0.41
187	102	34	*Phacelia tanacetifolia*	Annual	04.04.2024	4/5/24	NA	1	0.00	0.04
188	102	34	*Phacelia tanacetifolia*	Annual	04.04.2024	4/5/24	NA	2	0.00	0.02
189	102	34	*Phacelia tanacetifolia*	Annual	04.04.2024	4/7/24	NA	3	0.01	0.02
190	102	34	*Phacelia tanacetifolia*	Annual	04.04.2024	4/7/24	NA	4	0.00	0.01
191	102	34	*Phacelia tanacetifolia*	Annual	04.04.2024	4/7/24	NA	5	0.18	0.16
192	102	34	*Phacelia tanacetifolia*	Annual	04.04.2024	4/7/24	NA	6	0.00	0.00
193	104	35	*Phacelia tanacetifolia*	Annual	04.11.2024	4/17/24	Bottom	1	0.89	0.90
196	104	35	*Phacelia tanacetifolia*	Annual	04.11.2024	4/20/24	Bottom	4	0.63	0.61
195	104	35	*Phacelia tanacetifolia*	Annual	04.11.2024	4/19/24	Middle	3	0.50	0.49
198	104	35	*Phacelia tanacetifolia*	Annual	04.11.2024	4/22/24	Middle	6	0.54	0.56
194	104	35	*Phacelia tanacetifolia*	Annual	04.11.2024	4/18/24	Top	2	0.18	0.19
197	104	35	*Phacelia tanacetifolia*	Annual	04.11.2024	4/21/24	Top	5	0.08	0.12
67	5	2	*Calamintha nepeta*	Perennial	When purchased	4/5/24	NA	1	0.02	0.05
68	5	2	*Calamintha nepeta*	Perennial	When purchased	4/7/24	NA	2	0.00	0.08
69	5	2	*Calamintha nepeta*	Perennial	When purchased	4/14/24	NA	3	0.10	0.22

70	5	2	*Calamintha nepeta*	Perennial	When purchased	4/14/24	NA	4	0.00	0.06
71	5	2	*Calamintha nepeta*	Perennial	When purchased	4/15/24	NA	5	0.04	0.07
72	5	2	*Calamintha nepeta*	Perennial	When purchased	4/17/24	NA	6	0.14	0.16
73	17	6	*Calamintha nepeta*	Perennial	When purchased	4/5/24	NA	1	0.18	0.16
74	17	6	*Calamintha nepeta*	Perennial	When purchased	4/7/24	NA	2	0.33	0.45
75	17	6	*Calamintha nepeta*	Perennial	When purchased	4/14/24	NA	3	0.13	0.17
76	17	6	*Calamintha nepeta*	Perennial	When purchased	4/14/24	NA	4	0.15	0.19
77	17	6	*Calamintha nepeta*	Perennial	When purchased	4/15/24	NA	5	0.17	0.13
78	17	6	*Calamintha nepeta*	Perennial	When purchased	4/16/24	NA	6	0.25	0.21
79	25	9	*Calamintha nepeta*	Perennial	04.04.2024	4/5/24	NA	1	0.65	0.62
80	25	9	*Calamintha nepeta*	Perennial	04.04.2024	4/7/24	NA	2	0.25	0.35
81	25	9	*Calamintha nepeta*	Perennial	04.04.2024	4/14/24	NA	3	0.15	0.22
82	25	9	*Calamintha nepeta*	Perennial	04.04.2024	4/14/24	NA	4	0.08	0.15
83	25	9	*Calamintha nepeta*	Perennial	04.04.2024	4/15/24	NA	5	0.23	0.26
84	25	9	*Calamintha nepeta*	Perennial	04.04.2024	4/16/24	NA	6	0.28	0.23
85	46	16	*Calamintha nepeta*	Perennial	04.27.2024	4/27/24	NA	1	0.31	0.32
86	46	16	*Calamintha nepeta*	Perennial	04.27.2024	4/27/24	NA	2	0.05	0.09
87	46	16	*Calamintha nepeta*	Perennial	04.27.2024	4/30/24	NA	3	0.12	0.11
88	46	16	*Calamintha nepeta*	Perennial	04.27.2024	4/30/24	NA	4	0.07	0.06
89	46	16	*Calamintha nepeta*	Perennial	04.27.2024	5/5/24	NA	5	0.54	0.45
90	46	16	*Calamintha nepeta*	Perennial	04.27.2024	5/5/24	NA	6	0.28	0.35
103	80	27	*Calamintha nepeta*	Perennial	04.05.2024	4/5/24	NA	1	0.37	0.33
104	80	27	*Calamintha nepeta*	Perennial	04.05.2024	4/7/24	NA	2	0.40	0.49
105	80	27	*Calamintha nepeta*	Perennial	04.05.2024	4/14/24	NA	3	0.62	1.04
106	80	27	*Calamintha nepeta*	Perennial	04.05.2024	4/14/24	NA	4	0.32	0.16
107	80	27	*Calamintha nepeta*	Perennial	04.05.2024	4/15/24	NA	5	0.32	0.34
108	80	27	*Calamintha nepeta*	Perennial	04.05.2024	4/16/24	NA	6	0.09	0.07
109	81	27	*Calamintha nepeta*	Perennial	04.04.2024	4/5/24	NA	1	0.22	0.25
110	81	27	*Calamintha nepeta*	Perennial	04.04.2024	4/7/24	NA	2	0.14	0.07
111	81	27	*Calamintha nepeta*	Perennial	04.04.2024	4/14/24	NA	3	0.14	0.20
112	81	27	*Calamintha nepeta*	Perennial	04.04.2024	4/14/24	NA	4	0.26	0.44
113	81	27	*Calamintha nepeta*	Perennial	04.04.2024	4/15/24	NA	5	0.45	0.55
114	81	27	*Calamintha nepeta*	Perennial	04.04.2024	4/16/24	NA	6	0.03	0.11
115	90	30	*Calamintha nepeta*	Perennial	04.30.2024	4/30/24	NA	1	0.25	0.21
116	90	30	*Calamintha nepeta*	Perennial	04.30.2024	4/30/24	NA	2	0.52	0.46
117	90	30	*Calamintha nepeta*	Perennial	04.30.2024	4/30/24	NA	3	0.46	0.43
118	90	30	*Calamintha nepeta*	Perennial	04.30.2024	4/30/24	NA	4	0.01	0.03
119	90	30	*Calamintha nepeta*	Perennial	04.30.2024	4/30/24	NA	5	0.55	0.44
120	90	30	*Calamintha nepeta*	Perennial	04.30.2024	4/30/24	NA	6	0.00	0.02
121	106	36	*Calamintha nepeta*	Perennial	When purchased	4/5/24	NA	1	0.33	0.37

Supplementary information for: Interspecific variation in nectar and pollen reward among plant species commonly planted to support pollinators

122	106	36	*Calamintha nepeta*	Perennial	When purchased	4/7/24	NA	2	0.22	0.29
123	106	36	*Calamintha nepeta*	Perennial	When purchased	4/14/24	NA	3	0.36	0.30
124	106	36	*Calamintha nepeta*	Perennial	When purchased	4/14/24	NA	4	0.22	0.26
125	106	36	*Calamintha nepeta*	Perennial	When purchased	4/15/24	NA	5	0.20	0.26
126	106	36	*Calamintha nepeta*	Perennial	When purchased	4/16/24	NA	6	0.04	0.11
331	10	4	*Linaria purpurea*	Perennial	04.27.2024	4/27/24	Bottom	1	0.44	0.70
332	10	4	*Linaria purpurea*	Perennial	04.27.2024	4/27/24	Bottom	2	0.49	0.54
333	10	4	*Linaria purpurea*	Perennial	04.27.2024	4/27/24	Middle	3	0.35	0.38
334	10	4	*Linaria purpurea*	Perennial	04.27.2024	4/27/24	Middle	4	0.10	0.13
336	10	4	*Linaria purpurea*	Perennial	04.27.2024	4/27/24	Top	6	0.24	0.58
335	10	4	*Linaria purpurea*	Perennial	04.27.2024	4/27/24	Top	5	0.20	0.20
337	13	5	*Linaria purpurea*	Perennial	04.13.2024	4/24/24	Bottom	1	0.81	1.41
338	13	5	*Linaria purpurea*	Perennial	04.13.2024	4/24/24	Bottom	2	1.15	1.86
339	13	5	*Linaria purpurea*	Perennial	04.13.2024	4/24/24	Middle	3	0.39	0.53
340	13	5	*Linaria purpurea*	Perennial	04.13.2024	4/25/24	Middle	4	0.54	0.59
342	13	5	*Linaria purpurea*	Perennial	04.13.2024	4/27/24	Top	6	0.84	1.01
341	13	5	*Linaria purpurea*	Perennial	04.13.2024	4/27/24	Top	5	0.06	0.13
343	14	5	*Linaria purpurea*	Perennial	04.21.2024	4/14/24	Bottom	1	0.12	0.23
344	14	5	*Linaria purpurea*	Perennial	04.21.2024	4/14/24	Bottom	2	0.18	0.26
345	14	5	*Linaria purpurea*	Perennial	04.21.2024	4/24/24	Middle	3	0.78	0.63
346	14	5	*Linaria purpurea*	Perennial	04.21.2024	4/25/24	Middle	4	0.84	1.01
348	14	5	*Linaria purpurea*	Perennial	04.21.2024	4/27/24	Top	6	0.11	0.12
347	14	5	*Linaria purpurea*	Perennial	04.21.2024	4/27/24	Top	5	0.47	0.25
349	39	13	*Linaria purpurea*	Perennial	04.09.2024	4/24/24	Bottom	1	0.48	0.46
350	39	13	*Linaria purpurea*	Perennial	04.09.2024	4/24/24	Bottom	2	1.51	1.51
351	39	13	*Linaria purpurea*	Perennial	04.09.2024	4/24/24	Middle	3	0.35	0.36
352	39	13	*Linaria purpurea*	Perennial	04.09.2024	4/25/24	Middle	4	1.42	1.49
354	39	13	*Linaria purpurea*	Perennial	04.09.2024	4/27/24	Top	6	0.31	0.35
353	39	13	*Linaria purpurea*	Perennial	04.09.2024	4/27/24	Top	5	0.17	0.23
355	47	16	*Linaria purpurea*	Perennial	04.21.2024	4/24/24	Bottom	1	1.49	1.32
356	47	16	*Linaria purpurea*	Perennial	04.21.2024	4/24/24	Bottom	2	0.61	0.61
357	47	16	*Linaria purpurea*	Perennial	04.21.2024	4/24/24	Middle	3	0.27	0.23
358	47	16	*Linaria purpurea*	Perennial	04.21.2024	4/25/24	Middle	4	0.58	0.57
360	47	16	*Linaria purpurea*	Perennial	04.21.2024	4/27/24	Top	6	0.23	0.38
359	47	16	*Linaria purpurea*	Perennial	04.21.2024	4/27/24	Top	5	0.11	0.06
361	48	16	*Linaria purpurea*	Perennial	04.09.2024	4/14/24	Bottom	1	1.74	1.85
362	48	16	*Linaria purpurea*	Perennial	04.09.2024	4/14/24	Bottom	2	1.48	1.67
363	48	16	*Linaria purpurea*	Perennial	04.09.2024	4/24/24	Middle	3	0.14	0.37
364	48	16	*Linaria purpurea*	Perennial	04.09.2024	4/25/24	Middle	4	0.28	0.57
366	48	16	*Linaria purpurea*	Perennial	04.09.2024	4/27/24	Top	6	0.19	0.14

365	48	16	*Linaria purpurea*	Perennial	04.09.2024	4/27/24	Top	5	0.30	0.24
367	51	17	*Linaria purpurea*	Perennial	04.25.2024	4/24/24	Bottom	1	0.12	0.13
368	51	17	*Linaria purpurea*	Perennial	04.25.2024	4/25/24	Bottom	2	0.29	0.42
369	51	17	*Linaria purpurea*	Perennial	04.25.2024	4/25/24	Middle	3	0.01	0.07
370	51	17	*Linaria purpurea*	Perennial	04.25.2024	4/25/24	Middle	4	0.23	0.29
372	51	17	*Linaria purpurea*	Perennial	04.25.2024	4/27/24	Top	6	0.03	0.08
371	51	17	*Linaria purpurea*	Perennial	04.25.2024	4/27/24	Top	5	0.09	0.07
373	57	19	*Linaria purpurea*	Perennial	04.30.2024	4/30/24	Bottom	1	0.57	0.59
374	57	19	*Linaria purpurea*	Perennial	04.30.2024	4/30/24	Bottom	2	0.61	0.71
375	57	19	*Linaria purpurea*	Perennial	04.30.2024	4/30/24	Middle	3	0.48	0.49
376	57	19	*Linaria purpurea*	Perennial	04.30.2024	4/30/24	Middle	4	0.46	0.46
378	57	19	*Linaria purpurea*	Perennial	04.30.2024	4/30/24	Top	6	0.02	0.10
377	57	19	*Linaria purpurea*	Perennial	04.30.2024	4/30/24	Top	5	0.34	0.35
379	71	24	*Linaria purpurea*	Perennial	04.13.2024	4/14/24	Bottom	1	0.12	0.32
380	71	24	*Linaria purpurea*	Perennial	04.13.2024	4/24/24	Bottom	2	1.49	1.46
381	71	24	*Linaria purpurea*	Perennial	04.13.2024	4/24/24	Middle	3	1.05	1.41
382	71	24	*Linaria purpurea*	Perennial	04.13.2024	4/25/24	Middle	4	0.91	1.01
384	71	24	*Linaria purpurea*	Perennial	04.13.2024	4/27/24	Top	6	0.46	0.60
383	71	24	*Linaria purpurea*	Perennial	04.13.2024	4/27/24	Top	5	0.11	0.10
385	82	28	*Linaria purpurea*	Perennial	04.17.2024	4/14/24	Bottom	1	1.01	0.90
386	82	28	*Linaria purpurea*	Perennial	04.17.2024	4/14/24	Bottom	2	0.72	0.73
387	82	28	*Linaria purpurea*	Perennial	04.17.2024	4/24/24	Middle	3	0.43	0.40
388	82	28	*Linaria purpurea*	Perennial	04.17.2024	4/25/24	Middle	4	1.16	1.25
390	82	28	*Linaria purpurea*	Perennial	04.17.2024	4/27/24	Top	6	0.15	0.16
389	82	28	*Linaria purpurea*	Perennial	04.17.2024	4/27/24	Top	5	0.05	0.14
391	95	32	*Linaria purpurea*	Perennial	05.02.2024	5/5/24	Bottom	1	0.12	0.09
392	95	32	*Linaria purpurea*	Perennial	05.02.2024	5/5/24	Bottom	2	0.19	0.22
393	95	32	*Linaria purpurea*	Perennial	05.02.2024	5/5/24	Middle	3	0.52	0.58
394	95	32	*Linaria purpurea*	Perennial	05.02.2024	5/5/24	Middle	4	0.70	0.73
396	95	32	*Linaria purpurea*	Perennial	05.02.2024	5/5/24	Top	6	0.11	0.19
395	95	32	*Linaria purpurea*	Perennial	05.02.2024	5/5/24	Top	5	0.10	0.21
199	8	3	*Salvia rosmarinus*	Perennial	When purchased	4/7/24	NA	1	0.44	0.52
200	8	3	*Salvia rosmarinus*	Perennial	When purchased	4/7/24	NA	2	0.58	0.59
201	8	3	*Salvia rosmarinus*	Perennial	When purchased	4/7/24	NA	3	0.17	0.19
202	8	3	*Salvia rosmarinus*	Perennial	When purchased	4/9/24	NA	4	0.43	0.45
203	8	3	*Salvia rosmarinus*	Perennial	When purchased	4/9/24	NA	5	1.25	1.29
204	8	3	*Salvia rosmarinus*	Perennial	When purchased	4/9/24	NA	6	1.34	1.36
205	15	5	*Salvia rosmarinus*	Perennial	When purchased	4/7/24	NA	1	0.38	0.45
206	15	5	*Salvia rosmarinus*	Perennial	When purchased	4/7/24	NA	2	0.15	0.22
207	15	5	*Salvia rosmarinus*	Perennial	When purchased	4/7/24	NA	3	0.31	0.44

208	15	5	*Salvia rosmarinus*	Perennial	When purchased	4/9/24	NA	4	0.18	0.16
209	15	5	*Salvia rosmarinus*	Perennial	When purchased	4/9/24	NA	5	0.00	0.04
210	15	5	*Salvia rosmarinus*	Perennial	When purchased	4/9/24	NA	6	0.08	0.10
211	26	9	*Salvia rosmarinus*	Perennial	When purchased	4/7/24	NA	1	0.50	0.47
212	26	9	*Salvia rosmarinus*	Perennial	When purchased	4/7/24	NA	2	0.30	0.43
213	26	9	*Salvia rosmarinus*	Perennial	When purchased	4/7/24	NA	3	0.16	0.27
214	26	9	*Salvia rosmarinus*	Perennial	When purchased	4/14/24	NA	4	0.08	0.07
217	50	17	*Salvia rosmarinus*	Perennial	When purchased	4/7/24	NA	1	0.35	0.37
218	50	17	*Salvia rosmarinus*	Perennial	When purchased	4/7/24	NA	2	0.31	0.29
219	50	17	*Salvia rosmarinus*	Perennial	When purchased	4/7/24	NA	3	0.47	0.50
220	50	17	*Salvia rosmarinus*	Perennial	When purchased	4/9/24	NA	4	0.39	0.42
221	50	17	*Salvia rosmarinus*	Perennial	When purchased	4/9/24	NA	5	0.39	0.35
222	50	17	*Salvia rosmarinus*	Perennial	When purchased	4/9/24	NA	6	0.74	0.46
223	65	22	*Salvia rosmarinus*	Perennial	04.09.2024	4/7/24	NA	1	0.18	0.24
224	65	22	*Salvia rosmarinus*	Perennial	04.09.2024	4/7/24	NA	2	0.75	0.76
225	65	22	*Salvia rosmarinus*	Perennial	04.09.2024	4/7/24	NA	3	0.81	0.93
226	65	22	*Salvia rosmarinus*	Perennial	04.09.2024	4/9/24	NA	4	0.72	0.74
227	65	22	*Salvia rosmarinus*	Perennial	04.09.2024	4/9/24	NA	5	0.16	0.22
228	65	22	*Salvia rosmarinus*	Perennial	04.09.2024	4/9/24	NA	6	0.83	0.87
229	67	23	*Salvia rosmarinus*	Perennial	04.09.2024	4/7/24	NA	1	0.21	0.20
230	67	23	*Salvia rosmarinus*	Perennial	04.09.2024	4/7/24	NA	2	0.41	0.42
231	67	23	*Salvia rosmarinus*	Perennial	04.09.2024	4/7/24	NA	3	0.37	0.41
232	67	23	*Salvia rosmarinus*	Perennial	04.09.2024	4/9/24	NA	4	0.44	0.39
233	67	23	*Salvia rosmarinus*	Perennial	04.09.2024	4/9/24	NA	5	0.38	0.39
234	67	23	*Salvia rosmarinus*	Perennial	04.09.2024	4/9/24	NA	6	1.04	0.95
235	92	31	*Salvia rosmarinus*	Perennial	When purchased	4/7/24	NA	1	0.48	0.60
236	92	31	*Salvia rosmarinus*	Perennial	When purchased	4/7/24	NA	2	0.49	0.59
237	92	31	*Salvia rosmarinus*	Perennial	When purchased	4/7/24	NA	3	0.55	0.81
238	92	31	*Salvia rosmarinus*	Perennial	When purchased	4/9/24	NA	4	0.61	0.59
239	92	31	*Salvia rosmarinus*	Perennial	When purchased	4/9/24	NA	5	0.99	0.97
240	92	31	*Salvia rosmarinus*	Perennial	When purchased	4/9/24	NA	6	0.20	0.18
241	98	33	*Salvia rosmarinus*	Perennial	When purchased	4/7/24	NA	1	0.42	0.49
242	98	33	*Salvia rosmarinus*	Perennial	When purchased	4/7/24	NA	2	0.37	0.50
243	98	33	*Salvia rosmarinus*	Perennial	When purchased	4/7/24	NA	3	0.23	0.32
244	98	33	*Salvia rosmarinus*	Perennial	When purchased	4/9/24	NA	4	0.02	0.10
245	98	33	*Salvia rosmarinus*	Perennial	When purchased	4/9/24	NA	5	0.10	0.17
246	98	33	*Salvia rosmarinus*	Perennial	When purchased	4/9/24	NA	6	0.44	0.44
403	58	20	*Scrophularia californica*	Perennial	04.11.2024	4/18/24	Bottom	1	4.48	3.07
404	58	20	*Scrophularia californica*	Perennial	04.11.2024	4/18/24	Bottom	2	4.31	3.07
406	58	20	*Scrophularia californica*	Perennial	04.11.2024	4/30/24	Bottom	4	1.88	1.63

405	58	20	*Scrophularia californica*	Perennial	04.11.2024	4/18/24	Top	3	3.01	1.53
411	59	20	*Scrophularia californica*	Perennial	04.11.2024	4/18/24	Bottom	1	4.19	3.07
414	59	20	*Scrophularia californica*	Perennial	04.11.2024	4/30/24	Bottom	4	1.56	0.51
412	59	20	*Scrophularia californica*	Perennial	04.11.2024	4/18/24	Middle	2	3.60	3.07
413	59	20	*Scrophularia californica*	Perennial	04.11.2024	4/18/24	Top	3	2.67	2.29
419	62	21	*Scrophularia californica*	Perennial	04.11.2024	4/18/24	Bottom	1	1.33	1.12
420	62	21	*Scrophularia californica*	Perennial	04.11.2024	4/30/24	Bottom	2	3.49	2.54
421	62	21	*Scrophularia californica*	Perennial	04.11.2024	4/30/24	Middle	3	3.61	2.97
427	63	21	*Scrophularia californica*	Perennial	04.11.2024	4/18/24	Bottom	3	1.36	1.31
428	63	21	*Scrophularia californica*	Perennial	04.11.2024	4/30/24	Bottom	4	3.25	2.79
425	63	21	*Scrophularia californica*	Perennial	04.11.2024	4/18/24	Top	1	2.54	1.81
426	63	21	*Scrophularia californica*	Perennial	04.11.2024	4/18/24	Top	2	1.85	1.15
431	69	23	*Scrophularia californica*	Perennial	04.25.2024	4/30/24	Bottom	1	2.65	1.21
432	69	23	*Scrophularia californica*	Perennial	04.25.2024	4/30/24	Bottom	2	2.33	1.69
433	69	23	*Scrophularia californica*	Perennial	04.25.2024	4/30/24	Middle	3	3.35	2.63
434	69	23	*Scrophularia californica*	Perennial	04.25.2024	4/30/24	Middle	4	3.40	2.71
437	73	25	*Scrophularia californica*	Perennial	04.11.2024	4/18/24	Bottom	1	0.03	0.03
438	73	25	*Scrophularia californica*	Perennial	04.11.2024	4/18/24	Bottom	2	3.97	3.07
440	73	25	*Scrophularia californica*	Perennial	04.11.2024	4/30/24	Bottom	4	1.42	1.19
439	73	25	*Scrophularia californica*	Perennial	04.11.2024	4/18/24	Middle	3	4.03	3.07
445	87	29	*Scrophularia californica*	Perennial	04.07.2024	4/18/24	Bottom	1	4.49	3.07
446	87	29	*Scrophularia californica*	Perennial	04.07.2024	4/18/24	Bottom	2	2.43	2.24
447	87	29	*Scrophularia californica*	Perennial	04.07.2024	4/18/24	Middle	3	4.17	3.07
453	88	30	*Scrophularia californica*	Perennial	04.07.2024	4/18/24	Bottom	1	4.03	3.00
454	88	30	*Scrophularia californica*	Perennial	04.07.2024	4/18/24	Bottom	2	3.50	3.07
455	88	30	*Scrophularia californica*	Perennial	04.07.2024	4/18/24	Middle	3	4.14	3.07
462	100	34	*Scrophularia californica*	Perennial	04.17.2024	4/18/24	Bottom	1	4.31	3.08
463	100	34	*Scrophularia californica*	Perennial	04.17.2024	4/18/24	Bottom	2	4.03	3.07
471	105	35	*Scrophularia californica*	Perennial	04.11.2024	4/18/24	Bottom	2	3.86	3.07
470	105	35	*Scrophularia californica*	Perennial	04.11.2024	4/18/24	Middle	1	4.16	2.85
472	105	35	*Scrophularia californica*	Perennial	04.11.2024	4/18/24	Middle	3	3.77	3.07
1	9	3	*Verbena lilacina*	Perennial	When purchased	3/26/24	Umbel_1	1	0.11	0.07
2	9	3	*Verbena lilacina*	Perennial	When purchased	3/26/24	Umbel_2	2	0.53	0.14
3	9	3	*Verbena lilacina*	Perennial	When purchased	3/26/24	Umbel_3	3	0.10	0.05
4	9	3	*Verbena lilacina*	Perennial	When purchased	3/27/24	Umbel_4	4	0.34	0.32
5	9	3	*Verbena lilacina*	Perennial	When purchased	4/2/24	Umbel_5	5	0.03	0.08
6	9	3	*Verbena lilacina*	Perennial	When purchased	4/2/24	Umbel_6	6	0.32	0.23
7	16	6	*Verbena lilacina*	Perennial	When purchased	3/26/24	Umbel_1	1	0.31	0.12
8	16	6	*Verbena lilacina*	Perennial	When purchased	3/26/24	Umbel_2	2	0.19	0.11
9	16	6	*Verbena lilacina*	Perennial	When purchased	3/26/24	Umbel_3	3	0.09	0.02

10	16	6	*Verbena lilacina*	Perennial	When purchased	3/27/24	Umbel_4	4	0.00	0.07
11	16	6	*Verbena lilacina*	Perennial	When purchased	4/2/24	Umbel_5	5	0.11	0.03
12	16	6	*Verbena lilacina*	Perennial	When purchased	4/2/24	Umbel_6	6	0.11	0.05
13	22	8	*Verbena lilacina*	Perennial	When purchased	3/26/24	Umbel_1	1	0.15	0.05
14	22	8	*Verbena lilacina*	Perennial	When purchased	3/26/24	Umbel_2	2	0.08	0.09
15	22	8	*Verbena lilacina*	Perennial	When purchased	3/26/24	Umbel_3	3	0.15	0.05
16	22	8	*Verbena lilacina*	Perennial	When purchased	3/27/24	Umbel_4	4	0.14	0.14
17	22	8	*Verbena lilacina*	Perennial	When purchased	4/2/24	Umbel_5	5	0.04	0.02
18	22	8	*Verbena lilacina*	Perennial	When purchased	4/2/24	Umbel_6	6	0.22	0.08
19	29	10	*Verbena lilacina*	Perennial	When purchased	3/26/24	Umbel_1	1	0.00	0.00
20	29	10	*Verbena lilacina*	Perennial	When purchased	3/26/24	Umbel_2	2	0.06	0.04
21	29	10	*Verbena lilacina*	Perennial	When purchased	3/26/24	Umbel_3	3	0.34	0.21
22	29	10	*Verbena lilacina*	Perennial	When purchased	3/27/24	Umbel_4	4	0.35	0.14
23	29	10	*Verbena lilacina*	Perennial	When purchased	4/2/24	Umbel_5	5	0.00	0.00
24	29	10	*Verbena lilacina*	Perennial	When purchased	4/2/24	Umbel_6	6	0.09	0.02
25	36	12	*Verbena lilacina*	Perennial	When purchased	3/26/24	Umbel_1	1	0.31	0.11
26	36	12	*Verbena lilacina*	Perennial	When purchased	3/26/24	Umbel_2	2	0.25	0.14
27	36	12	*Verbena lilacina*	Perennial	When purchased	3/26/24	Umbel_3	3	0.21	0.03
28	36	12	*Verbena lilacina*	Perennial	When purchased	3/27/24	Umbel_4	4	0.46	0.37
29	36	12	*Verbena lilacina*	Perennial	When purchased	4/2/24	Umbel_5	5	0.13	0.04
30	36	12	*Verbena lilacina*	Perennial	When purchased	4/2/24	Umbel_6	6	0.31	0.20
31	66	22	*Verbena lilacina*	Perennial	When purchased	3/26/24	Umbel_1	1	1.22	0.28
32	66	22	*Verbena lilacina*	Perennial	When purchased	3/26/24	Umbel_2	2	0.30	0.19
33	66	22	*Verbena lilacina*	Perennial	When purchased	3/26/24	Umbel_3	3	0.67	0.31
34	66	22	*Verbena lilacina*	Perennial	When purchased	3/27/24	Umbel_4	4	0.14	0.07
35	66	22	*Verbena lilacina*	Perennial	When purchased	4/2/24	Umbel_5	5	0.13	0.09
36	66	22	*Verbena lilacina*	Perennial	When purchased	4/2/24	Umbel_6	6	0.49	0.35
37	68	23	*Verbena lilacina*	Perennial	When purchased	3/26/24	Umbel_1	1	0.32	0.20
38	68	23	*Verbena lilacina*	Perennial	When purchased	3/26/24	Umbel_2	2	0.10	0.07
39	68	23	*Verbena lilacina*	Perennial	When purchased	3/26/24	Umbel_3	3	0.79	0.43
40	68	23	*Verbena lilacina*	Perennial	When purchased	3/27/24	Umbel_4	4	0.39	0.35
41	68	23	*Verbena lilacina*	Perennial	When purchased	4/2/24	Umbel_5	5	0.03	0.03
42	68	23	*Verbena lilacina*	Perennial	When purchased	4/2/24	Umbel_6	6	0.34	0.25
43	75	25	*Verbena lilacina*	Perennial	When purchased	3/26/24	Umbel_1	1	0.09	0.11
44	75	25	*Verbena lilacina*	Perennial	When purchased	3/26/24	Umbel_2	2	0.15	0.14
45	75	25	*Verbena lilacina*	Perennial	When purchased	3/26/24	Umbel_3	3	0.00	0.08
46	75	25	*Verbena lilacina*	Perennial	When purchased	3/27/24	Umbel_4	4	0.57	0.57
47	75	25	*Verbena lilacina*	Perennial	When purchased	4/2/24	Umbel_5	5	0.16	0.11
48	75	25	*Verbena lilacina*	Perennial	When purchased	4/2/24	Umbel_6	6	0.41	0.10
49	86	29	*Verbena lilacina*	Perennial	When purchased	3/26/24	Umbel_1	1	0.24	0.11

50	86	29	*Verbena lilacina*	Perennial	When purchased	3/26/24	Umbel_2	2	0.50	0.45
51	86	29	*Verbena lilacina*	Perennial	When purchased	3/26/24	Umbel_3	3	0.80	0.34
52	86	29	*Verbena lilacina*	Perennial	When purchased	3/27/24	Umbel_4	4	0.71	0.39
53	86	29	*Verbena lilacina*	Perennial	When purchased	4/2/24	Umbel_5	5	0.33	0.24
54	86	29	*Verbena lilacina*	Perennial	When purchased	4/2/24	Umbel_6	6	0.66	0.55
55	91	31	*Verbena lilacina*	Perennial	When purchased	3/26/24	Umbel_1	1	0.02	0.09
56	91	31	*Verbena lilacina*	Perennial	When purchased	3/26/24	Umbel_2	2	0.27	0.18
57	91	31	*Verbena lilacina*	Perennial	When purchased	3/26/24	Umbel_3	3	0.37	0.26
58	91	31	*Verbena lilacina*	Perennial	When purchased	3/27/24	Umbel_4	4	0.11	0.06
59	91	31	*Verbena lilacina*	Perennial	When purchased	4/2/24	Umbel_5	5	0.37	0.21
60	91	31	*Verbena lilacina*	Perennial	When purchased	4/2/24	Umbel_6	6	0.40	0.25
61	99	33	*Verbena lilacina*	Perennial	When purchased	3/26/24	Umbel_1	1	0.29	0.17
62	99	33	*Verbena lilacina*	Perennial	When purchased	3/26/24	Umbel_2	2	0.46	0.33
63	99	33	*Verbena lilacina*	Perennial	When purchased	3/26/24	Umbel_3	3	0.43	0.37
64	99	33	*Verbena lilacina*	Perennial	When purchased	3/27/24	Umbel_4	4	0.11	0.04
65	99	33	*Verbena lilacina*	Perennial	When purchased	4/2/24	Umbel_5	5	0.20	0.11
66	99	33	*Verbena lilacina*	Perennial	When purchased	4/2/24	Umbel_6	6	0.03	0.03
